# LIF regulates CXCL9 in tumor-associated macrophages and prevents CD8^+^ T cell tumor-infiltration impairing anti-PD1 therapy

**DOI:** 10.1038/s41467-019-10369-9

**Published:** 2019-06-11

**Authors:** Mónica Pascual-García, Ester Bonfill-Teixidor, Ester Planas-Rigol, Carlota Rubio-Perez, Raffaella Iurlaro, Alexandra Arias, Isabel Cuartas, Ada Sala-Hojman, Laura Escudero, Francisco Martínez-Ricarte, Isabel Huber-Ruano, Paolo Nuciforo, Leire Pedrosa, Carolina Marques, Irene Braña, Elena Garralda, María Vieito, Massimo Squatrito, Estela Pineda, Francesc Graus, Carmen Espejo, Juan Sahuquillo, Josep Tabernero, Joan Seoane

**Affiliations:** 1 Vall d Hebron Institute of Oncology (VHIO), Vall d’Hebron University Hospital, 08035 Barcelona, Spain; 2 CIBERONC, 028029 Madrid, Spain; 3 Vall d’Hebron Institut de Recerca (VHIR), Vall d’Hebron University Hospital, 08035 Barcelona, Spain; 4 Universitat Autònoma de Barcelona, 08193 Cerdanyola del Vallès, Spain; 5 Hospital Clinic, University of Barcelona and Institut d’Investigació Biomèdica August Pi i Sunyer (IDIBAPS), 08036 Barcelona, Spain; 6 Seve Ballesteros Foundation Brain Tumor Group, Spanish National Cancer Research Center, CNIO, 28029 Madrid, Spain; 7 Institució Catalana de Recerca i Estudis Avançats (ICREA), 08010 Barcelona, Spain

**Keywords:** Cancer, Cancer microenvironment

## Abstract

Cancer response to immunotherapy depends on the infiltration of CD8^+^ T cells and the presence of tumor-associated macrophages within tumors. Still, little is known about the determinants of these factors. We show that LIF assumes a crucial role in the regulation of CD8^+^ T cell tumor infiltration, while promoting the presence of protumoral tumor-associated macrophages. We observe that the blockade of LIF in tumors expressing high levels of LIF decreases CD206, CD163 and CCL2 and induces CXCL9 expression in tumor-associated macrophages. The blockade of LIF releases the epigenetic silencing of CXCL9 triggering CD8^+^ T cell tumor infiltration. The combination of LIF neutralizing antibodies with the inhibition of the PD1 immune checkpoint promotes tumor regression, immunological memory and an increase in overall survival.

## Abstract (web summary)

LIF is a pleiotropic cytokine that promotes an immunosuppressive microenvironment and has critical functions in embryonic development. Here, the authors show that LIF regulates CD8^+^ T cell tumor infiltration in cancer by repressing CXCL19 and promoting the presence of protumoral macrophages and thatLIF inhibition, via neutralizing antibodies, promotes T cell infiltration and synergizes with immune checkpoint inhbitors resulting in tumor regression and immunological memory.

## Introduction

LIF is a pleiotropic cytokine with a critical dual function in embryonic development^1,2^. LIF regulates embryonic stem cell (ESC) self-renewal preventing early differentiation^3^ and is required for blastocyst implantation in the uterus^4^. Specifically, LIF generates a local immunosuppressive microenvironment in order to protect the embryo from the mother’s immune system^4–7^. Ablation of LIF in mice prevents embryo implantation, and women with loss-of-function LIF mutations show infertility with impairment of in vitro fertilization^4–7^. Thus, LIF is a master regulator of embryo implantation through the regulation of the immune tolerance in pregnancy.

LIF can act as an oncogenic factor through the induction of the self-renewal of cancer-initiating cells^8^, the regulation of cancer-associated fibroblasts^9^, as well as promoting radioresistance^10^ and chemoresistance^11^. Importantly, the expression of LIF in cancer is widely heterogeneous and some tumors express aberrantly high levels of LIF while others do not express LIF^12^. Non-small cell lung cancer, breast cancer, colorectal cancer, and nasopharyngeal cancer patients with tumors expressing high levels of LIF exhibit a shorter overall survival^8–10^ indicating that high LIF expression might be involved in the oncogenic progression of these malignancies. However, the molecular mechanisms underlying the role of LIF in the immune response in cancer have not been elucidated yet.

Here, we find that tumors expressing high levels of LIF tend to be infiltrated with tumor-associated macrophages (TAMs). LIF regulates the expression of CD163 and CD206, as well as several chemokines including CXCL9 and CCL2 in TAMs. Specifically, LIF triggers the epigenetic silencing of the CXCL9 locus by the recruitment of EZH2 to the CXCL9 promoter. The blockade of LIF in tumors expressing high levels of LIF releases the CXCL9 repression acting as a chemoattractant of cytotoxic CD8^+^ T cells. LIF is then a crucial regulator of CD8^+^ T cell tumor infiltration. Importantly, the combination of the blockade of LIF with checkpoint inhibitors induces tumor regression, immune memory, and an increase in overall survival.

## Results

### LIF expression correlates with TAM tumor infiltration

**Fig. 1:**
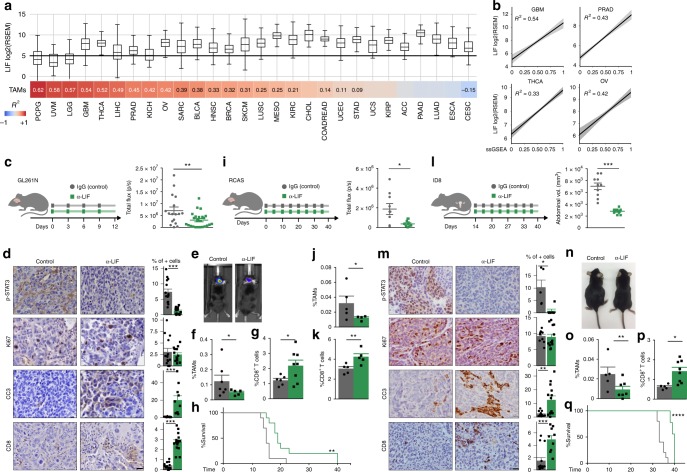
LIF blockade decreases tumor growth and regulates immune cell infiltration. **a** Top-panel, distribution of LIF mRNA expression (log2 RSEM) across 28 distinct solid tumors (see Supplementary Data 1b) in boxplots (middle line depicts the median and the whiskers the interquartile range). Black line represents the estimated cut-off between low expression/background noise. Bottom panel, correlation values (Pearson *R*^2^ values) between LIF expression and the relative abundance of TAMs based on ssGSEA of the gene signature (see Supplementary Data 1a). Correlation values are only shown if the correlation is significant (adjusted *P*-value < 0.1) (see Supplementary Data 1c). Cancer types are sorted according correlation value between LIF and TAMs relative abundance. **b** Linear regression plots of LIF expression and relative abundance (ssGSEA rescaled from 0 to 1 for visualization purposes) of TAMs in GBM, prostate adenocarcinoma (PRAD), thyroid carcinoma (THCA) and ovarian carcinoma (OV) cohorts. Shade represents the confidence intervals of the regression estimate. **c**, **i**, **l** Tumor growth of GL261N (**c**), RCAS (**i**), and ID8 (**l**) models measured as total flux (p/s) or abdominal volume (mm^3^), respectively. Scheme representing the experimental procedure is shown. Anti-LIF or isotype control (IgG) treatment started on the day of surgery (GL261N and RCAS) or 14 days post-inoculation (dpi) (ID8). **d**, **m** Representative p-STAT3, Ki67, CC3, and CD8 IHC images and percentages of staining for GL261N (**d**) and ID8 (**m**) tumors. **e**, **n** Representative images of GL261N (**e**) and ID8 (**n**) tumor-bearing mice treated with anti-LIF or IgG. **f**–**g**, **j**–**k**, **o**–**p**, Percentages of CD11b^+^ F4/80^+^ CD163^+^ CD206^+^ MHCII^low^ TAMs (**f**, **o**) or CD11b^+^ Ly6G^−^ Ly6C^−^ CD163^+^ CD206^+^ MHCII^low^ (**j**) and CD8^+^ T cells (CD3^+^ CD8^+^) of GL261N (**g**), RCAS (**k**), and ID8 (**p**) tumors analysed by flow cytometry. **h**, **q**, Overall survival of GL261N (**h**) and ID8 (**q**) models treated with anti-LIF or IgG. Data are mean ± SEM. Statistical analyses by Mann–Whitney *T*-test and Log-rank test. *P < 0.05; **P < 0.01; ***P < 0.001; ****P < 0.0001

To study the effect of LIF on the cancer immune system, we first determined whether LIF expression correlated with the presence of tumor immune cell infiltrates in human cancer. We observed a significant correlation between LIF and TAMs across several tumor types of The Cancer Genome Atlas (TCGA) (Fig. 1a, b, Supplementary Data 1). Glioblastoma (GBM), prostate adenocarcinoma, thyroid cancer and ovarian cancer were the 4 tumor types exhibiting the highest correlations between LIF and TAMs, while showing a high LIF expression across tumor samples (Fig. 1a, b). A wide range of LIF expression was observed in GBM tumors being it expressed by tumor cells and the immune cell infiltrates (Supplementary Fig. 1).

### The blockade of LIF induces an antitumor immune response

We decided to use immunocompetent mouse models of GBM and ovarian cancer (tumor types where LIF strongly correlates with TAMs) to study the potential immune-modulating role of LIF in cancer. We identified the GBM cell line, GL261N (a derivative of the GL261 cell line), the *GFAP-tv-a* RCAS-PDGFA, shp53, shNF1 (RCAS) transgenic model^13^ and the ovarian cancer cell line, ID8, that generated tumors in the brain (GL261N and RCAS) and peritoneum (ID8) of mice expressing high levels of LIF (Supplementary Fig. 2a).

We repressed LIF function in GL261N, RCAS and ID8 models using neutralizing antibodies, CRISPR/CAS9 or RNA interference technologies and observed a decrease in tumor growth and a modest increase in survival (Fig. 1c, e, h, i, l, n, q, Supplementary Figs. 2b–f,  3e, f). The blockade of LIF in the GL261 tumor model, a tumor that did not express LIF, did not inhibit tumor growth (Supplementary Fig. 2a, g).

Neutralizing antibodies against LIF induced a marked decrease in p-STAT3 levels showing that in these animal models (selected based on high LIF expression) LIF was the main cytokine inducing the JAK-STAT3 pathway (Fig. 1d, m). Moreover, while we did not observe a significant decrease in Ki67 positive cells, we detected an increase in cleaved caspase 3 (CC3) indicating that the blockade of LIF induced tumor cell death (Fig. 1d, m).

In order to evaluate the role of the immune system in the response to anti-LIF treatment, we performed experiments using immunodeficient animals. Treatment of GL261N tumors in RAG^−/−^ or NOD SCID mice (both strains of mice lacking the adaptive immune response) with anti-LIF did not show a significant impact on tumor growth (Supplementary Fig. 2h). This indicated that in our models the antitumor response to the blockade of LIF was mainly mediated by the adaptive immune response.

We decided to further investigate the molecular mechanisms involved in the immune response to anti-LIF treatment. We observed a decrease in the number of protumoral TAMs (Fig. 1f, j, o) and, importantly, a concomitant increase in tumor infiltration of CD8^+^ T cells upon anti-LIF treatment (Fig. 1d, g, k, m, p). Natural killer (NK) and regulatory T (Treg) cell numbers increased and decreased upon treatment with anti-LIF, respectively (Supplementary Fig. 2i–l). Infiltrating CD8^+^ T cells expressed Granzyme A (GZMA) suggesting that they were mediating the cytotoxic effect (Supplementary Fig. 3a). Moreover a compartment of CD8^+^ T cells expressed PD1 (Supplementary Fig. 3b, c). TAMs derived from recruited monocytes (CD11b^+^ Ly6G^−^ Ly6C^−^ CD49d^+^)^14^ were decreased in response to anti-LIF or LIF shRNA (Supplementary Fig. 3d–f) and no major effect was observed on the dendritic cell population (CD11b^+^, CD11c^+^, MHCII^+^) (Supplementary Fig. 3g) nor on the levels of IL12 or IL10 in the tissue (Supplementary Fig. 3h).

We then assessed whether the LIF-mediated regulation of the tumor immune infiltrates was the cause or the consequence of the antitumor response. To this end, we performed an acute treatment experiment where we treated mice with established tumors with anti-LIF for 4 days. The 4 day-treatment did not affect tumor growth (Supplementary Fig. 3i) but was enough to engage CD8^+^ T cell tumor infiltration (Supplementary Fig. 3j). This showed that CD8^+^ T cell infiltration was not the result of the anti-tumor response to the blockade of LIF.

### LIF regulates the expression of protumoral cytokines in TAMs

**Fig. 2:**
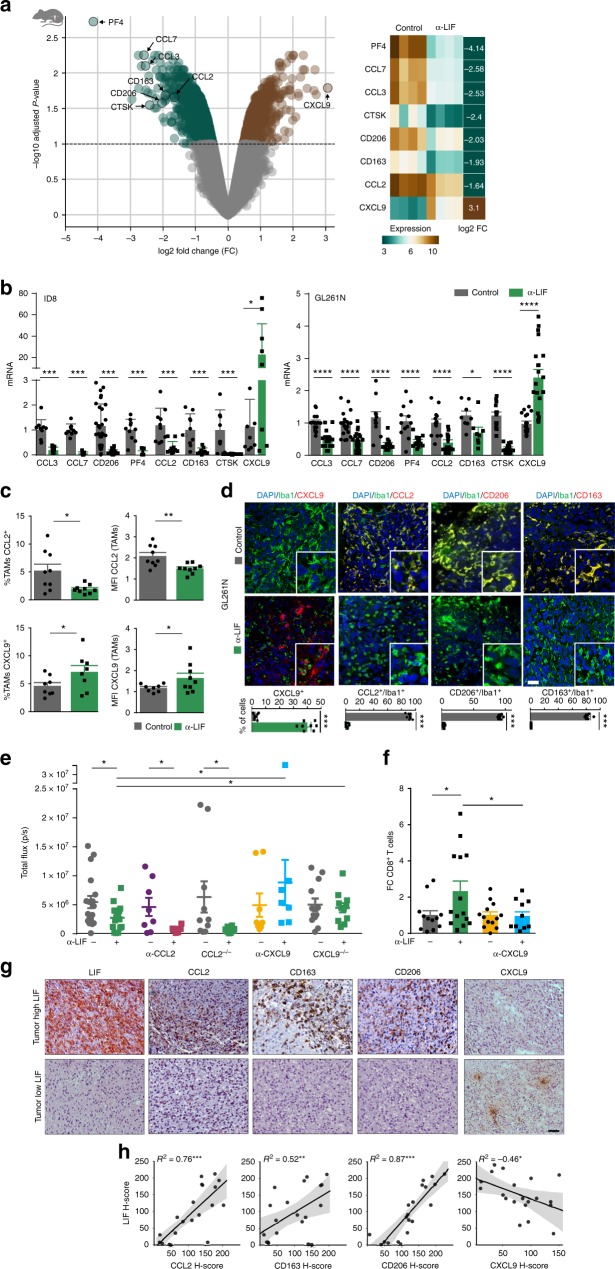
LIF regulates CXCL9, CCL2, CD206, and CD163 in TAMs. **a** Differential expression analysis of isolated CD11b^+^ cells from anti-LIF treated ID8 mice vs. control. Volcano plot representing the genes significantly (*Q*-value < 0.1) overexpressed (brown) and significantly underexpressed (turquoise). Heatmap representing the expression values of the indicated genes, each column represents a sample and each row a gene. The last column represents the log2 fold change (log2 FC) of gene expression. **b** mRNA expression for the indicated genes in isolated CD11b^+^ cells from anti-LIF treated or untreated ID8 and GL261N tumors. **c** Percentage and mean fluorescence intensity (MFI) of CCL2 and CXCL9 in TAMs (CD11b^+^ Ly6G^−^ Ly6C^−^) from anti-LIF treated or untreated GL261N tumors. **d** Representative IF images of Iba1 and the indicated markers stainings of GL261N tumors (see Supplementary Fig. 4). Scale bar, 20 μm. Bottom, percentage of double positive cells relative to the TAM marker positive cells. CXCL9 quantification is relative to the total number of cells. **e** Tumor growth of GL261N in WT, CXCL9^−/−^, and CCL2^−/−^ mice or mice treated with the indicated antibodies is shown as total flux (p/s). **f** Fold change (FC) of tumor infiltrating CD8^+^ T cells in the indicated treatments. Data are mean ± SEM. **g**, **h** Representative IHC of the indicated markers from 20 GBM tumors. The degree of staining was quantified using *H*-score method. Correlations between LIF and CCL2, CD206, CD163, and CXCL9 with the *R*-squared coefficients (*R*^2^) were calculated (**h**). Statistical analyses by Mann–Whitney *T*-test. *P < 0.05; **P < 0.01; ***P < 0.001; ****P < 0.0001

We isolated CD11b^+^ cells from the ID8 mouse model treated or untreated with anti-LIF antibodies and performed a transcriptomic analysis. Several genes related to an oncogenic phenotype were downregulated (i.e., CCL2, CCL3, CCL7, PF4, CTSK, CD206, CD163) and, interestingly, CXCL9 was upregulated (Fig. 2a, Supplementary Data 2). The aforementioned gene responses were validated by qRT-PCR in the ID8 and GL261N models (Fig. 2b).

CXCL9 and CCL2 stood out as chemokines described to be critical for CD8^+^ T cell tumor infiltration, and the recruitment of TAMs and Tregs^15–17^, respectively. We confirmed the CXCL9 and CCL2 regulation in TAMs (CD11b^+^ Ly6G^−^ Ly6C^−^) by the neutralization of LIF or LIF shRNA (Fig. 2c, Supplementary Fig. 3k–m). Immunofluorescence (IF) and isolation of TAMs showed that CXCL9, CCL2, CD206, and CD163 were mainly expressed in TAMs (Fig. 2d, Supplementary Fig. 4, 5a) and inhibition of LIF function regulated their expression (Fig. 2c, d, Supplementary Figs. 3k–m,  4). CXCR3 (CXCL9 receptor), CCR2 (CCL2 receptor) and LIFR were expressed in TAMs and CD8^+^ T cells (Supplementary Fig. 5b).

In order to address the relevance of the regulation of CXCL9 and CCL2 in the LIF oncogenic function, we used CXCL9 and CCL2 knockout (CXCL9^−/−^, CCL2^−/−^) mouse models, and blocking antibodies against CXCL9 and CCL2. Interestingly, the anti-tumor response to the inhibition of LIF was blunted in the CXCL9^−/−^ mice but not in the CCL2^−/−^ mice (Fig. 2e). Similarly, the CXCL9 neutralizing antibody but not the CCL2 antibody impaired the anticancer response to anti-LIF (Fig. 2e). These results indicated that the main mediator of the anti-LIF response was CXCL9. As expected, the blockade of CXCL9 prevented the increase of CD8^+^ T cell tumor infiltration in response to anti-LIF (Fig. 2f). We then investigated whether the TAM-specific expression of CXCL9 was responsible for the regulation of the tumor infiltration of CD8^+^ T cells. We generated GL261N tumors in CXCL9^−/−^ and CCL2^−/−^ mice and hence the tumors were infiltrated with CXCL9^−/−^ or CCL2^−/−^ TAMs. We then transplanted the tumors into wild type mice and assessed CD8^+^ T cell tumor infiltration in response to the blockade of LIF. We observed that the TAM-specific deletion of CXCL9 reduced the infiltration of CD8^+^ T cells in response to the blockade of LIF (Supplementary Fig. 5c, d). In contrast, the TAM-specific deletion of CCL2 did not impact on the CD8^+^ T cell tumor recruitment (Supplementary Fig. 5c, d).

We went back to the analysis of TCGA datasets of human GBM and ovarian cancer and observed a significantly positive correlation between LIF and CCL2, CD163, and CD206 in both tumor types (Supplementary Fig. 6). No correlation was observed between LIF and CXCL9. In order to validate these results at the protein level, we analysed a cohort of 20 GBM patients and performed LIF, CXCL9, CCL2, CD163, and CD206 IHC of the tumors. We noted a strong positive correlation between LIF and CCL2, CD163 and CD206 (Fig. 2g, h). CXCL9 was expressed in isolated clusters of cells and its levels showed an inverse correlation with LIF in human GBM (Fig. 2g, h).

### LIF induces the epigenetic silencing of CXCL9

**Fig. 3:**
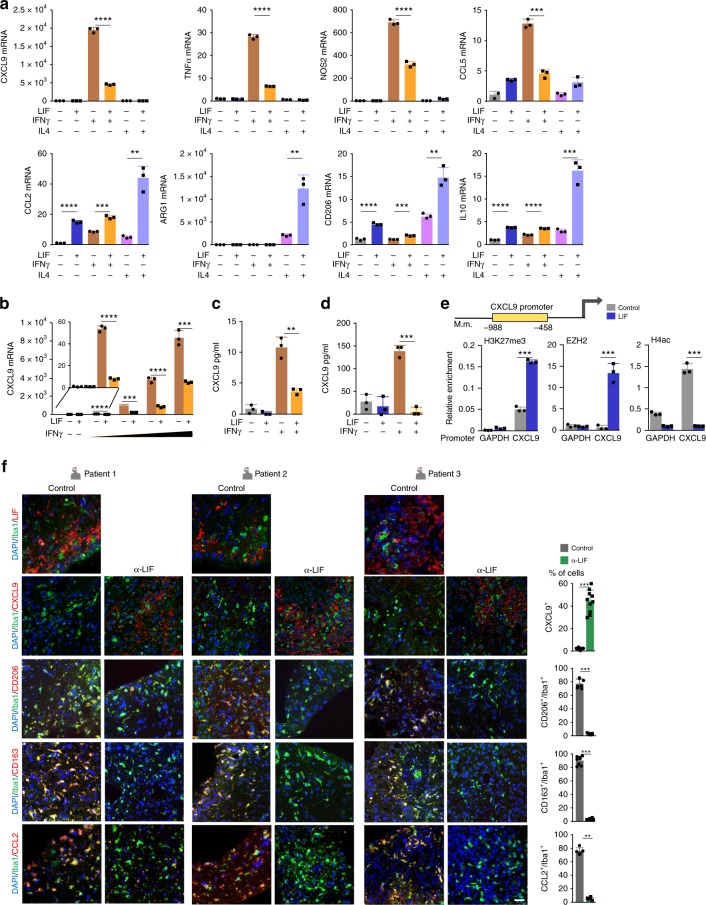
LIF represses CXCL9 through epigenetic silencing. **a**, **b** qRT-PCR analysis of the indicated genes in BMDMs. BMDMs were pre-incubated with 20 ng/ml LIF for 72 h and then stimulated with 5 ng/ml IFNγ or 10 μg/ml IL4 during 6 h (**a**) or with 0.1, 0.5, 1 and 5 ng/ml IFNγ for 24 h (**b**). **c** CXCL9 ELISA from BMDMs pre-incubated with 20 ng/ml LIF and then stimulated with 0.1 ng/ml IFNγ for 24 h. **d** CXCL9 ELISA from human CD11b^+^sorted cells (77% CD11b^+^ CD14^+^, see Supplementary Fig. 7b, c) from human GBM cultured with 20 ng/ml LIF for 72 h and then with 0.1 ng/ml IFNγ for 24 h. **e** ChIP of Tri-methyl-histone H3 (H3K27me3), EZH2 and acetyl-histone4 (H4ac) was performed in BMDMs treated with 20 ng/ml LIF for 72 h. Scheme shows the analysed CXCL9 promoter region. Representative data are presented as mean ± SD. **f** Representative images of IF of the indicated markers in human GBM organotypic slices (patients 1, 2, 3) incubated with 10 μg/ml anti-LIF for 3 days. Scale bar, 20 μm. (see Supplementary Fig. 8). Bottom, percentage of double positive cells relative to Iba1^+^ cells and percentage of CXCL9^+^ cells relative to the total number of cells. Data are mean of all patients ± SEM. Statistical analyses by Student’s *t*-test or Mann–Whitney *T*-test. *P < 0.05, **P < 0.01; ***P < 0.001; ****P < 0.0001

We decided to explore the molecular mechanisms involved in the regulation of CXCL9 by LIF in macrophages. To this end, we studied the effect of LIF on primary cultures of mouse bone marrow derived macrophages (BMDMs). LIF regulated the expression of several M1-like and M2-like markers induced by IFNγ or IL4 in BMDMs (Fig. 3a). CXCL9 expression was not detected except when BMDMs were treated with IFNγ. Recombinant LIF repressed the induction of CXCL9 by IFNγ both at the mRNA or protein levels (Fig. 3b, c). A similar result was observed when BMDMs were treated with LPS (Supplementary Fig. 7a). Importantly, CXCL9 was also regulated by IFNγ and LIF in patient-derived TAMs (CD11b^+^ CD14^+^) obtained from fresh human GBM tumors (Fig. 3d, Supplementary Fig. 7b, c). Thus, LIF acted as a repressor of CXCL9 induction. CXCL9 has been described to undergo epigenetic regulation^18^ and LIF is known to regulate the epigenetic status of embryonic stem cells^1,19^. In line with this, we found that treatment with LIF increased the levels of H3 lysine 27 trimethylated (H3K27me3), decreased the levels of acetylated H4 (H4ac), and increased EZH2 binding to the CXCL9 promoter region, altogether showing that LIF was regulating CXCL9 expression through epigenetic silencing (Fig. 3e).

### LIF regulates CD8^+^ T cell tumor infiltration

To confirm that LIF regulates immune cell tumor infiltration through the repression of CXCL9 in tumors from actual cancer patients, we generated organotypic tissue cultures of GBM specimens freshly obtained from patients. These organotypic models allow for the short-term culture of tumor slices that maintain the tissue architecture and stroma (including immune cells) of the patient’s tumor^20^. We obtained organotypic tissue cultures from three patient-derived tumors expressing high levels of LIF (Fig. 3f, Supplementary Fig. 8). In all three cultures a large infiltration of TAMs was present as detected by the Iba1 marker and most of the TAMs expressed CCL2, CD163, and CD206. Interestingly, a 3-day treatment of the organotypic culture with a neutralizing antibody against LIF promoted a decrease in CCL2, CD163, and CD206 and an increase in CXCL9 expression (Fig. 3f, Supplementary Fig. 8).

**Fig. 4:**
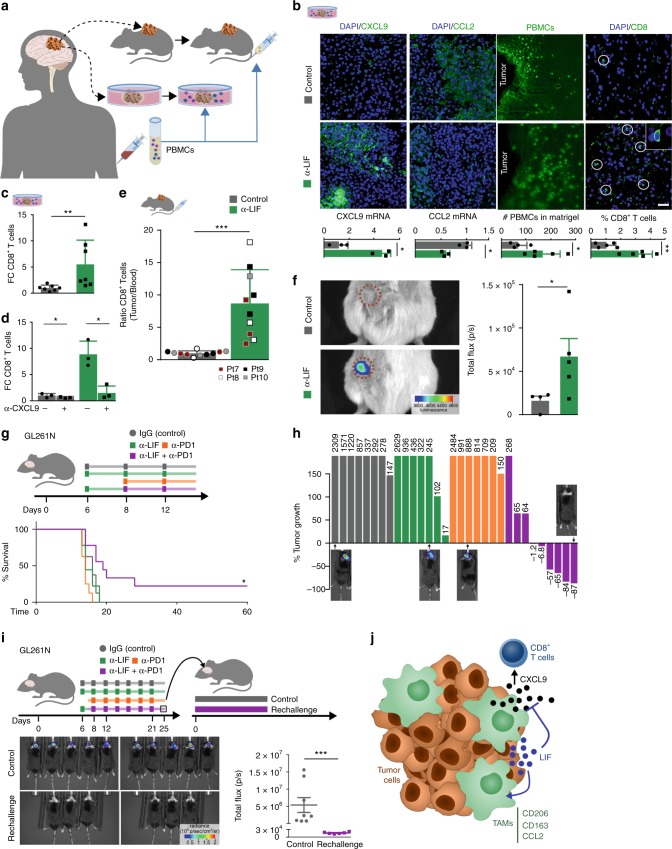
LIF blockade induces CD8^+^ T cell tumor infiltration and promotes tumor regression when combined with anti-PD1. **a** Schematic representation of GBM patient-derived xenografts and human organotypic models. **b** Organotypic specimens were treated with anti-LIF for 72 h and then cultured with PBMCs for 24 h. CXCL9 and CCL2 mRNA expression levels are shown. Representative images (patient 4, 5, 6) of CFSE-stained PBMCs into Matrigel containing GBM specimens and IF of the indicated factors in organotypic tissues are displayed. Bars represent quantification of five different fields of each condition. Data are presented as mean ± SD. Scale bar, 20 μm. **c**, **d** FC of CD8^+^ T infiltrating cells detected by flow cytometry in organotypic tissues treated with anti-LIF (**c**, **d**) and/or anti-CXCL9 (1.5 μg/ml) (**d**) for 72 h and then cultured with PBMCs for 48 h (patient 4, 5, 6). **e** CD8^+^ T infiltrating cells into subcutaneously engrafted GBM specimens in NSG mice. Bar graph represents the ratio of CD8^+^ T cells detected by flow cytometry in the tissue vs. CD8^+^ T cells detected in the blood of the same animal. Four patients (7, 8, 9, 10) with their corresponding PBMCs were evaluated and are represented with different colors. **f** Representative images of treated or untreated patient-derived xenografts (PDX) bearing mice 48 h after luciferase CD3^+^ T cells inoculation. T cells infiltration is measured as total flux (p/s) within the tumor. Data are mean ± SEM. **g** Scheme represents the experimental procedure of GL261N mice treated with anti-LIF, anti-PD1 or the combination. Overall survival determined by Kaplan-Meier curves is shown. **h** Tumor growth of the treated GL261N model represented as a fold change of tumor size between 13 and 6 dpi. Representative images are shown. **i** Scheme representing the experimental procedure for regression study. Images of mice and tumor size measured by total flux (p/s) at 13 dpi are shown. **j** Schematic representation shows the effect of LIF in CD8^+^ T cell tumor infiltration. Statistical analyses by Mann–Whitney *T*-test or Log-rank test. *P < 0.05; **P < 0.01; ***P < 0.001

We then decided to test whether the regulation of LIF could impact on the immune cell tumor infiltration. After anti-LIF treatment, we incubated the organotypic slices from three patient-derived tumors (expressing high levels of LIF) with peripheral blood mononuclear cells (PBMCs) from the same patient (Fig. 4a). Treatment with anti-LIF increased CXCL9 and decreased CCL2 expression (Fig. 4b), while inducing immune cell infiltration into the Matrigel surrounding the tumor specimen (Fig. 4b). Notably, CD8^+^ T cells were recruited to the tumor tissue upon LIF blockade (Fig. 4b, c, Supplementary Fig. 9) and this effect was dependent on CXCL9, since its neutralization prevented CD8^+^ T cell infiltration (Fig. 4d).

We next decided to confirm this result in the context of an in vivo model. We inoculated tumor fragments from four patients, whose tumors expressed high LIF levels, in NSG mice and treated these mice with the LIF neutralizing antibody for 5 days. Next, we inoculated each patient’s PBMCs in the mice and analyzed the short-term tumor infiltration of T cells. Note that this experimental model cannot be used for long experiments, and hence survival analysis, due to graft-versus-host disease. Interestingly, mice treated with anti-LIF showed an increase in CD8^+^ T cell tumor infiltration and most of the infiltrating CD8^+^ T cells expressed the CXCL9 receptor, CXCR3 (Fig. 4e, Supplementary Fig. 10a, b). We also isolated CD3^+^ T cells from PBMCs and infected them with lentiviruses encoding luciferase. Interestingly, most of the cells expressed CXCR3. We inoculated these cells in mice bearing patient-derived tumors and were able to monitor the anti-LIF-mediated CD3^+^ T cell tumor infiltration by bioluminescence (Fig. 4f, Supplementary Fig. 10c, d)

### LIF and PD1 blockade promotes tumor regression

So far, our data indicated that the inhibition of LIF induced a change in the phenotype of TAMs increasing the expression of CXCL9 and leading to the recruitment of CD8^+^ T cells to the tumor. As expected based on the literature^21,22^, we observed that a subpopulation of CD8^+^ T cells within the tumor in our mouse models were PD1 positive (see Supplementary Fig. 3b, c). This indicated that the blockade of PD1 in this context might increase the anti-tumor response to anti-LIF agents. We decided to use our immunocompetent mouse models bearing overt tumors with anti-LIF and anti-PD1 antibodies and observed that the combination of the blockade of LIF and PD1 further decreased tumor growth when compared to each individual treatment. Moreover and importantly, the combined treatment with anti-LIF and anti-PD1 increased overall survival and induced tumor regression (Fig. 4g, h).

We collected the mice exhibiting complete tumor regression and reinoculated 3 × 10^5^ tumor cells. No tumor appeared in these mice while tumors rapidly grew in naive mice inoculated in parallel with the same number of cells (Fig. 4i). The result of this rechallenge experiment indicated that the combined treatment with anti-LIF and anti-PD1 generated immunological memory.

## Discussion

CD8^+^ T tumor infiltration and TAMs determine among other things the response to cancer immunotherapy^17,23–27^. We have observed that LIF acts as an oncogenic factor through the regulation of the immune system. LIF establishes a cross-talk between the innate and the adaptive immune response since LIF neutralization regulates the expression of a chemokine program in TAMs, including CXCL9, leading to an increase in CD8^+^ T cell tumor infiltration (Fig. 4j). This indicates that LIF is responsible for CD8^+^ T cell tumor exclusion and that it could be involved in resistance to immune checkpoint blockade. Indeed, we explored transcriptomic data of checkpoint inhibitor clinical trials^28^ and observed that LIF, and the genes that we found regulated by LIF, were associated to resistance to checkpoint inhibitor therapy (Supplementary Fig. 11).

The immunosuppressive response to LIF is not only seen in cancer. LIF has been shown to inhibit the immune response in organ transplantation^29^, autoimmune diseases^30^ as well as in embryo implantation^2,6^. As in many other cases, cancer hijacks a normal developmental program for its own gain. LIF has been designed through evolution to protect the embryo from the immune system of the mother and prevent early differentiation of ESCs. Tumors abduce this dual mechanism of action by secreting aberrantly high levels of LIF. In this way, LIF protects tumors from the host immune response and, as we and others have previously reported^8,31^, prevents differentiation of cancer-initiating cells.

Our work reveals a promising therapeutic target in cancer. The blockade of LIF in tumors expressing high levels of LIF promotes effector T cell tumor infiltration transforming a T cell excluded tumor into an inflamed tumor. This effect synergizes with checkpoint inhibitors inducing tumor regression in very aggressive tumors such as GBM. Therapies directed against LIF may therefore have broad applications in the treatment of cancer.

## Methods

### Patients

Human GBM specimens were obtained from the Vall d’Hebron University Hospital and Clinic Hospital. The clinical protocol was approved by the Vall d’Hebron Institutional Review Board and Clinic Hospital (CEIC), with informed consent obtained from all subjects.

### Human tumoroid and organotypic slice cultures

GBM tumoroids were generated as follows^8^. Briefly, tumor samples were processed within 30 min after surgical resection. Minced pieces of human GBM samples were digested with 200 U/ml collagenase I (Sigma) and 500 U/ml DNase I (Sigma) in PBS for 1 h at 37 °C with constant vigorous agitation. The single-cell suspension was filtered through a 70 μm cell strainer (BD Falcon) and washed with PBS. Finally, cells were resuspended and subsequently cultured in GBM medium that consisted of Neurobasal medium supplemented with B27, penicillin/streptomycin (all from Life Technologies) and growth factors (20 ng/ml EGF and 20 ng/ml FGF-2 (PeproTech)). GBM organotypic slice cultures were generated as follows. After resection, surgical specimens were cut with a scalpel into rectangular blocks of 5–10 mm length and 1–2 mm width and individually transferred into 0.4 µm membrane culture inserts (Millipore) within 6-well plates. Before placing the inserts into 6-well plates, 1.2 ml of Neurobasal medium (Life Technologies) supplemented with B27 (Life Technologies), penicillin/streptomycin (Life Technologies) and growth factors (20 ng/ml EGF and 20 ng/ml FGF-2) (PeproTech) were placed into each well. The cultures were kept at 37 °C with constant humidity, 95% air and 5% CO^2^. After one day, slices were treated with 10 µg/ml of a rat anti-mouse/human LIF blocking antibody (referred to as anti-LIF) (developed in house) or with its corresponding normal IgG (10 µg/ml) for 3 days. For the blocking CXCL9 studies, a neutralizing mouse monoclonal antibody against human CXCL9 (R&D Systems; MAB392) was added to the culture at 1.5 µg/ml. In some occasions, 0.1 ng/ml of human rIFNγ (R&D Systems) was added for 24 h. In parallel, peripheral blood mononuclear cells (PBMCs) were obtained from the whole blood of the same patient by centrifuge density separation using Lymphosep (Biowest). PBMCs were cryopreserved in RPMI medium supplemented with 10% inactivated FBS and 10% DMSO until use. For immune cell infiltration assays, control or anti-LIF slices were embedded into growth factor deprived Matrigel (Corning) with subsequent addition of 1 × 10^6^ PBMCs into 24-well plate in complete (10% inactivated FBS) RPMI medium. In addition, supernatants were collected and organotypic slices were recovered from Matrigel and further processed for IF and flow cytometry. In some conditions, PBMCs were resuspended with PBS at a concentration of 1 × 10^6^ cells/ml and incubated for 20 min with 5 µM Cell Trace CFSE (Invitrogen). After the incubation, cells were washed with RPMI and added to the sections embedded into Matrigel. After 24 h, fluorescent PBMCs invasion into Matrigel was evaluated under microscope by counting migrating cells in five different areas per each condition.

### Animals and in vivo experiments

All animal experiments were approved by and performed according to the guidelines of the Institutional Animal Care Committee of the Vall d’Hebron Research Institute in agreement with the European Union and national directives. Female C57BL/6 and NOD/SCID were purchased from Janvier, RAG^−/−^, CCL2^−/−^ and CXCL9^−/−^ from Jackson Laboratories and NOD scid gamma (NSG) from Charles River. For brain tumor models, 3 × 10^5^ GL261N, GL261 or RCAS cells, all of them with luciferase expression, were stereotactically inoculated into the corpus striatum of the right brain hemisphere (1 mm anterior and 1.8 mm lateral to the lambda; 2.5 mm intraparenchymal) of 8-week-old C57BL/6 mice. For ovary tumor model, 5 × 10^6^ ID8 ovarian cancer cells were intraperitoneally injected into 8-week-old C57BL/6 mice. A dose of 300 µg (ID8) or 600 µg (GL261N, GL261 and RCAS) of anti-LIF or a control IgG was administered intraperitoneally twice a week. Additionally, a dose of 200 µg of rat anti-mouse PD1 blocking antibody (anti-PD1, BioXCell; BE0146), anti-mouse/human/rat CCL2 antibody (MCP-1, BioXcell; BE0185) or 3 µg of anti-mouse CXCL9 antibody (R&D; AF-492-NA) was administered intraperitoneally twice a week. Tumor progression was monitored by body weight and by abdominal girth (ID8), or bioluminescence measurements using the Xenogen IVIS® Spectrum (GL261N, GL261, and RCAS). Mice were euthanized when they exhibited clinical signs of disease or distress (i.e., cachexia, anorexia, or increased respiratory rates) or when tumors began to interfere with normal body functions.

For brain tumor transplantation, GL261N tumors were isolated from tumor-bearing mice, embedded in pieces into Matrigel and subcutaneously implanted in new control or anti-LIF treated mice.

For regression study, 3 × 10^5^ GL261N cells were inoculated in 6 tumor bearing-mice with complete regression after anti-LIF, anti-PD1 combination treatment. Ten naive mice were inoculated 3 × 10^5^ GL261N cells in parallel.

### Primary cell cultures and cell lines

BMDMs were obtained from 6 to 10-week-old C57BL/6 mice^32^. Briefly, bone marrow precursors were cultured in DMEM (Life Technologies), supplemented with 20% heat inactivated FBS and 30% L-cell conditioned medium (cm) as a source of macrophage-colony stimulating factor. Differentiated macrophages were obtained after 6 days culture. L-cell cm was obtained from L929 cells grown in DMEM supplemented with 10% heat-inactivated FBS (Life Technologies). Human macrophages were isolated from human GBM specimens. Briefly, tumor tissue was enzymatically digested with Tumor Dissociation kit and CD11b^+^ cells were isolated using CD11b magnetic beads and the MultiMACS Cell24 separator Plus (all from Miltenyi Biotec). CD11b^+^ cells obtained were cultured in RPMI medium supplemented with 10% heat-inactivated FBS (Life Technologies). Recombinant LIF, IFNγ, LPS, and IL4 were purchased from Millipore, R&D Systems, Sigma and Creative BioMart, respectively.

GL261N were derived from GL261 cells that were obtained from Charles River and cultured in RPMI (Life Technologies). ID8 was a generous gift from Dr. George Coukos, Ludwing Institute for Cancer Research, Lausanne, and cultured in DMEM. The media for both cell lines were supplemented with 10% FBS and penicillin/streptomycin (Life Technologies). RCAS GFAP-tva PDGFA, shp53, shNF1 (RCAS) cells^13^ were cultured in Neurocult basal medium supplemented with Neurocult proliferation supplement, heparin, FGF, and EGF (all from StemCell Technologies).

### GBM patient-derived in vivo models

Surgical human GBM specimens were cut with a scalpel into rectangular blocks of 5–10 mm length and 1–2 mm width and transferred into a tube containing 100 µl of Matrigel (Corning) supplemented with 5 ng/ml of rIFNγ (R&D Systems). Each specimen was introduced subcutaneously into one or two flanks of 4 to 5-weeks-old NSG mice. Half of the mice received anti-LIF (600 µg) treatment every three days starting at the day of the implantation of the samples. Minimum of three specimens per condition were implanted. After 5 days, 1 × 10^7^ PBMCs obtained from the same patient, and previously stimulated with 150 U/ml of rIL2 (R&D Systems) for 24 h, were intravenously inoculated into the mice in exception of one (control without PBMCs). Forty-eight hours post-inoculation, engrafted tumors were collected and processed to determine CD8^+^ T cells infiltration by flow cytometry. Total blood of the mice was also processed using Ficoll-Paque Premium (Ge Healthcare) in order to obtain the PBMCs.

For immune cell infiltration assay, 1 × 10^7^ of luciferase expressing CD3^+^ T cells were intravenously introduced into control or anti-LIF (600 µg) treated GBM patient-derived models. After 48 h CD3^+^ T cells recruitment into the tumor was monitored by bioluminescence measurements using the Xenogen IVIS® Spectrum.

### Tumor digestion, flow cytometry, and cell isolation

Mice were euthanized and tumors were isolated. GL261N and RCAS tumors were enzymatically digested with Brain Tumor Dissociation kit and myelin was removed with Myelin Removal Beads II (all from Miltenyi Biotec). ID8 tumors were processed with Mouse Tumor Dissociation kit (Miltenyi Biotec) and ascitic liquids were collected. Human GBM specimens of the organotypic model and the patient-derived xenografts were enzymatically digested with Human Tumor Dissociation kit (Miltenyi Biotec).

From GL261N cell suspension, CD11b^+^ cells isolation was performed using anti-Ly6C-APC and anti-APC microbeads and anti Ly6G microbeads to deplete Ly6G^+^ and Ly6C^+^ populations and then with CD11b magnetic beads. CD45^+^ cells isolation was performed with anti-mouse CD45 magnetic beads. From ID8 cell suspension, CD11b^+^ cells were isolated using anti-CD11b magnetic beads. Finally, from organotypic slices, CD45^+^ cells isolation was performed using anti-human CD45 magnetic beads. All the isolation procedures were performed using the MultiMACS Cell24 separator Plus following manufacturer instructions and magnetic beads were purchased from Miltenyi Biotec.

The murine antibodies against CD3 (17-0032-82; 1:40), CD4 (11-0042-81; 1:250), CD335 (25-3351-82; 1:20), CD163 (12-1631-82; 1:40, MHC class II (47-5321-82; 1:80), CXCR3 (12-1831-82; 1:40) from eBioscience, CD45 (BD-550994; 1:200), CD8 (BD-557654; 1:40), F4/80 (BD-565410; 1:25), CD11b (BD-553310; 1:100), CD11c (BD-550261; 1:100), CD206 (BD-565250; 1:30), CD49d (BD-740012; 1:30) from BD Bioscience, LIFR (Novus FAB5990N; 1:20) from Novus Biologicals, Ly6G (127622; 1:200), Ly6C (128018; 1:150), CCR2 (150604; 1:50) and PD1 (135208; 1:80) from Biolegend were used for flow cytometry. Intracellular staining of FoxP3 (12-5773-82; 1:40), Granzyme A (GZMA) (48-5831-82; 1:80), CXCL9 (12-3009-80; 1:40) from eBioscience and CCL2 (505910; 1:100; Biolegend) were performed using a specific staining set (eBioscience). For flow human studies, antibodies against CD11b (333142; 1:100), CD14 (555397; 1:100) from BD Bioscience, CD45 (304032; 1:100), CD3 (344816; 1:100), CXCR3 (353708; 1:100) from Biolegend and CD8 (560179; 1:75; BD Pharmigen) were used. In some occasions samples were previously incubated with LIVE/DEAD fixable yellow dead stain kit (L34959; 1:2000;Thermo Fisher Scientific) to determine viability. As a positive control of human GBM organotypic and patient derived xenografts, 1 × 10^6^ PBMCs (named as “spike PBMCs”) was added into the control samples in order to establish the leukocyte populations.

Samples were acquired on a BD LSRFortessa™ cell analyser or Navios (Beckman Coulter) and data were analysed with Flow Jo software.

### Lentiviral infection

293T cells were transfected using lipofectamine 2000 (Invitrogen) transfection method with pMD2.G enveloping plasmid, psPAX.2 packaging plasmid, and either pLenti CMV Luciferase (v-168-1) (Addgene), pLKO.1 or pLKO.1-shLIF murine (Sigma). After 16 h, medium was replaced by fresh medium and lentivirus was harvested for an additional 24 h. For cell lines infection, medium containing recombinant lentivirus was added to cells with polybrene (Sigma) at a concentration of 8 µg/ml. Following 16 h of incubation, cells were washed and incubated in fresh medium previous to selection.

CD3^+^ T cells were negatively isolated from PBMCs using Dynabeads Untouched Human T cell Kit (Thermo Fisher). Isolated CD3^+^ T cells were infected with lentivirus vectors^33^. Briefly, a 24 well plate was pre-coated for 1 h at 37 °C with 1 µg/ml of anti-CD3 (clone OKT3, Thermo Fisher Scientific) and 5 µg/ml of CH-296 (Retronectin recombinant fibronectin fragment, Takara). CD3^+^ T cells were resuspended at concentration of 0.7 × 10^6^ cells/ml with RPMI supplemented with 10% heat-inactivated FBS and 600 IU/ml of rIL2 (R&D systems). 0.4 ml of cell suspension was mixed with 1 ml of luciferase lentiviral vector (supplemented with 600 IU/ml of rIL2 and 8 µg/ml of polybrene) and seeded into anti-CD3/CH-296 coated 24-well plate. Plate was centrifuged at 1000×*g* for 30 min at 32 °C. The day after, medium was replaced by fresh RPMI supplemented with 10% heat-inactivated FBS and 600 IU/ml of rIL2. After 4 days cells were subcultured 1:2 into new anti-CD3/CH-296 pre-coated 24-well plate until used. At same time point, luciferase was determined by the luciferase assay system (Promega).

### CRISPR/LIF cell line generation

Cells were transfected with the commercial mouse LIF CRISPR/Cas9 KO plasmid (Santa Cruz Biotechnology) following manufacturer’s specifications. Green Fluorescent Protein (GFP) was used as a selective label. After 3 days, transfected cells were selected by sorting. CRISPR/LIF-KO positive cells were checked by qRT-PCR and ELISA.

### ELISA

For the quantitative determination of CXCL9 and CCL2 protein levels secreted to the media, and IL10 and IL12 protein levels from tumor tissue, we used the Mouse or Human Duo-Set ELISA kit (R&D systems) following manufacturer’s specifications.

### Quantitative real-time PCR

Cells were lysed for mRNA extraction (RNeasy Mini or Micro Kit, Qiagen), retrotranscription (iScript Reverse Supermix from BioRad for mRNA), and qRT-PCR was performed using Taqman probes from Applied Biosystems, according to manufacturer’s recommendations. For paraffin-embedded sections, RNA was obtained by using High Pure FFPET RNA isolation kit (Roche) and following manufacturer instructions. Reactions were carried out in a CFX384 Touch™ Real-Time PCR Detection System (Bio-Rad) and results were expressed as fold change calculated by the Ct method relative to the control sample. Murine or human *ACTB* or *GAPDH* were used as internal normalization controls.

### Immunohistochemical and immunofluorescence staining

Immunohistochemical (IHC) staining was performed as follows. Slides were deparaffinized and hydrated. Antigen retrieval was performed using pH 6 or pH 9 Citrate Antigen Retrieval Solution (DAKO), 10 min 10% peroxidase (H_2_O_2_) and blocking solution (2% BSA) for 1 h at room temperature. As a detection system, EnVision FLEX + (DAKO) was used according to the manufacturer’s instructions, followed by counterstaining with hematoxilin, dehydration and mounting (DPX). The quantification of LIF, CCL2, CD163, CD206, and CXCL9 staining in GBM tumors from patients was expressed as *H*-score (3× percentage of strong staining + 2× percentage of moderate staining + percentage of weak staining), giving a range of 0–300. Quantification of p-STAT3, Ki67, CC3, and CD8 was performed with ImageJ, counting the total number of cells of three different fields per mouse, five mice/group, and calculating the percentage of positive cells. Data in graphs are presented as mean ± SEM.

IHC antibodies: human LIF (Atlas; HPA018844; 1:200), murine LIF (Atlas; HPA018844; 1:300), murine p-STAT3 (Cell Signaling; 9131; 1:50), murine Ki67 (AbCam; ab15580; 1:200), murine Cleaved-Caspase3 (CC3) (Cell Signaling; 9661; 1:500), murine CD8 (Bioss; bs-0648R; 1:200), human/murine CCL2 (Novus Biologicals;NBP2-22115; 1:200), human CXCL9 (Thermo Fischer Scientific; 701117; 1:100), human CD206 (Abcam; ab64693; 1:4000) and human CD163 (Leica Novacastra; NCL-L-CD163; 1:200).

Immunofluorescence (IF) staining was performed as follows. Nuclei were counterstained with DAPI and images were captured using a laser scanning confocal NIKON Eclipse Ti microscope. Quantification of immunofluorescence were performed with ImageJ, counting all or up to 100 cells positive for CD11b, Iba1, or CD3 of 2–3 different fields of each mouse, 3–5 mice/group, and calculating the percentage of those cells positive for CCL2, CD206, and CD163 inside the Iba1 positive population. For CXCL9, it was calculated the percentage of cells surrounded by the signal of this cytokine inside the total population of cells. For organotypic slices, 3–4 fields of each patient (*n* = 3) were quantified. For organotypic tissue immunofluorescence, five different Z-stack images per condition were processed with Fiji-Image J software. For CD8^+^ T cells, percentage of CD8^+^ T cells was calculated among the total population. Data in graphs are represented as mean ± SEM.

IF antibodies: human/murine CCL2 (Novus Biologicals; NBP-22115; 1:200), human/murine Iba1 (Wako; 019-19741; 1:1000 and Abcam; ab5076; 1:50), human/murine CD206 (Abcam; ab64693; 1:500), murine CD163 (Abcam; ab199402; 1:200), CXCL9 (murine R&D; AF-492-NA; 1:200; human Thermo Fischer Scientific; 701117; 1:200), human CD8 (DAKO; M7103; 1:200), human LIF (Atlas; HPA018844; 1:200) and human CD163 (Leica Novocastra; NCL-L-CD163; 1:200).

### Microarray expression analysis

RNA was assayed on the Affymetrix microarray platform with the Mouse Gene 2.1 ST. Next, it was normalized based on a Robust-Microarray Average (RMA)^34^ (by Progenika). We identified the genes differentially expressed in anti-LIF treated mice through a Bayesian linear regression considering paired samples, using *limma* Bioconductor package^35^.

### Chromatin immunoprecipitation assays

Immunoprecipitation of chromatin was performed according to the Upstate (Millipore) standard protocol. Briefly, 1.2 × 10^7^ BMDMs were fixed using 1% formaldehyde for 10 min at 37 °C, harvested, and sonicated to generate chromatin fragments of 200–500 bp. Then 20 µg of sheared chromatin was immunoprecipitated overnight with 2 µg of anti-Tri-Methyl-Histone H3 (Lys27) (Cell Signaling; 97335), anti-acetyl-H4 (Millipore; 06–866) or anti-EZH2 (Millipore; CS203195) antibody. Immunocomplexes were recovered using 20 µl of protein G magnetic beads, washed, and eluted. Cross-linking was reversed at 65 °C 4 h and immunoprecipitated DNA was recovered using the PCR purification kit from Qiagen. Genomic regions of interest were identified by real-time quantitative PCR (qPCR) using SYBR Green Master Mix (Invitrogen). Each value was corrected by the corresponding input chromatin sample. *CXCL9* promoter was amplified using “CCCCGTTGCAATACTTTCAT” (forward) and “CCCCGTTGCAATACTTTCAT” (reverse) primers (EZH2, acetyl-H4) or “TGCTGTTGAATGCCACTTTC” (forward) and “TCCCTGCTACCTTTTCCAGA” (reverse) primers (H3me3). Mouse *GAPDH* promoter (Diagenode) was amplified as a control.

### TCGA data sets analysis

We downloaded RNA-seq data from The Cancer Genome Atlas (TCGA) for 9,403 patients suffering from 28 distinct solid tumors (see details on the tumor cohorts considered in Supplementary Data 1b) from Firebrowse server (http://firebrowse.org, version 2016_01_28). The RNA-seq normalized data (RSEM) was log2 transformed for all downstream analyses. Next, we obtained the gene signatures of TAMs from ^37^ (see Supplementary Data 1a). Based on previous work^36–38^, we inferred the relative abundance of the immune populations through a single sample gene set enrichment (ssGSEA)^39^ across all patients. Thereafter we computed the correlation between LIF expression and the ssGSEA scores of the four immune populations and the correlation between LIF and a set of genes of interest (see Statistical tests).

### Transcriptome analysis of metastatic urothelial carcinoma anti-PD1 treated patients analysis

We downloaded RNA-seq from the phase 2 clinical trial (IMvigor210) which investigates the clinical activity of PD-L1 blockade with atezolizumab in metastatic urothelial carcinoma^28^ We downloaded RNA-seq data (FASTQ files) and clinical annotations from the European Genome-Pheno Archive (EGAS00001002556). We normalized RNA-seq counts into Transcripts per million (TPM) for Homo Sapiens GRCh38 using Kallisto^40^. Two groups of patients, as previously defined by the authors, were considered for downstream analyses based on their clinical response to atezolizumab: non-responders (patients with Progressive Disease and Sustained Disease after treatment) and responders (patients with Partial and Complete response after treatment). To compare the expression levels of LIF between both groups TPMs were log2 transformed. Next, we performed an enrichment analysis of the anti-LIF down-regulated genes in anti-PD1 non-responder patients. We considered as anti-LIF down-regulated genes those genes with a log fold change (FC) <−1.5 in anti-LIF treated ID8 mice (see Microarray expression analysis above). Then, we performed a pre-ranked Gene Set Enrichment Analysis^41^, using *gseapy* Python3 package, on the genes differentially expressed between non-responder and responder patients (we considered the log FC as rank). Ranks were computed using *rankdata* Python3 SciPy package^16^.

### Statistical analysis

Data were analysed using GraphPad Prism 5.0 software. To compare two different groups we calculated *P*-value (*P*) using Student’s *T*-test (paired or unpaired) for parametric variables and Mann–Whitney test for non-parametric variables. Survival curves comparison was performed using Log-rank (Mantel-Cox) test. *P < 0.05; **P < 0.01; ***P < 0.001; ****P < 0.0001. TCGA data was analysed with Python3 SciPy library^16^, the correlation coefficient (*R*^2^) was obtained using a Pearson’s correlation. *P*-values were adjusted for multiple testing correction based on a Benjamini-Hochberg False Discovery Rate.

### Reporting summary

Further information on research design is available in the Nature Research Reporting Summary linked to this article.

## Supplementary information

Supplementary Information

Supplementary Data 1

Supplementary Data 2

Reporting Summary

Description of Additional Supplementary Files

## Data Availability

The microarray data of the mice ovary tumor model has been deposited in the Gene Expression Omnibus (GEO)/NCBI public database (accession no. GSE79852).
