# Supplementary material for: LIF regulates CXCL9 in tumor-associated macrophages and prevents CD8+ T cell tumor-infiltration impairing anti-PD1 therapy
Source: Nat Commun. 2019 Jun 11;10:2416. doi: 10.1038/s41467-019-10369-9 (PMC6559950; doi:10.1038/s41467-019-10369-9)
Supplement: Supplementary file 1 — Supplementary Information [file 41467_2019_10369_MOESM1_ESM.pdf]

SUPPLEMENTARY MATERIAL

SUPPLEMENTARY FIGURES **1**

Supplementary Figure 1. LIF expression in tumors. 1

Supplementary Figure 2. LIF blockade in syngeneic models inhibits tumor growth. 2

Supplementary Figure 3. Characterization of the immune cell infiltrates upon treatment with anti-LIF. 5

Supplementary Figure 4. Regulation of CXCL9, CCL2, CD163 and CD206 expression by LIF in GL261N tumors. 7

Supplementary Figure 5. Cell specific expression of cytokines and receptors, and functional contributions. 9

Supplementary Figure 6. Correlation between LIF and CD163, CD206 and CCL2 expression in GBM and ovarian cancer. 10

Supplementary Figure 7. Regulation of CXCL9 by LIF in murine and human macrophages. 11

Supplementary Figure 8. Regulation of CXCL9, CCL2, CD163 and CD206 expression by LIF in human GBM tumors. 12

Supplementary Figure 9. LIF regulates infiltration of CD8+ T cells into human GBM organotypic slice cultures. 13

Supplementary Figure 10. LIF regulates infiltration of CD8+ T cells into human GBM specimens in vivo. 16

Supplementary Figure 11. LIF is associated to anti-PD1 treatment resistance in clinical trials. 17

SUPPLEMENTARY FIGURES

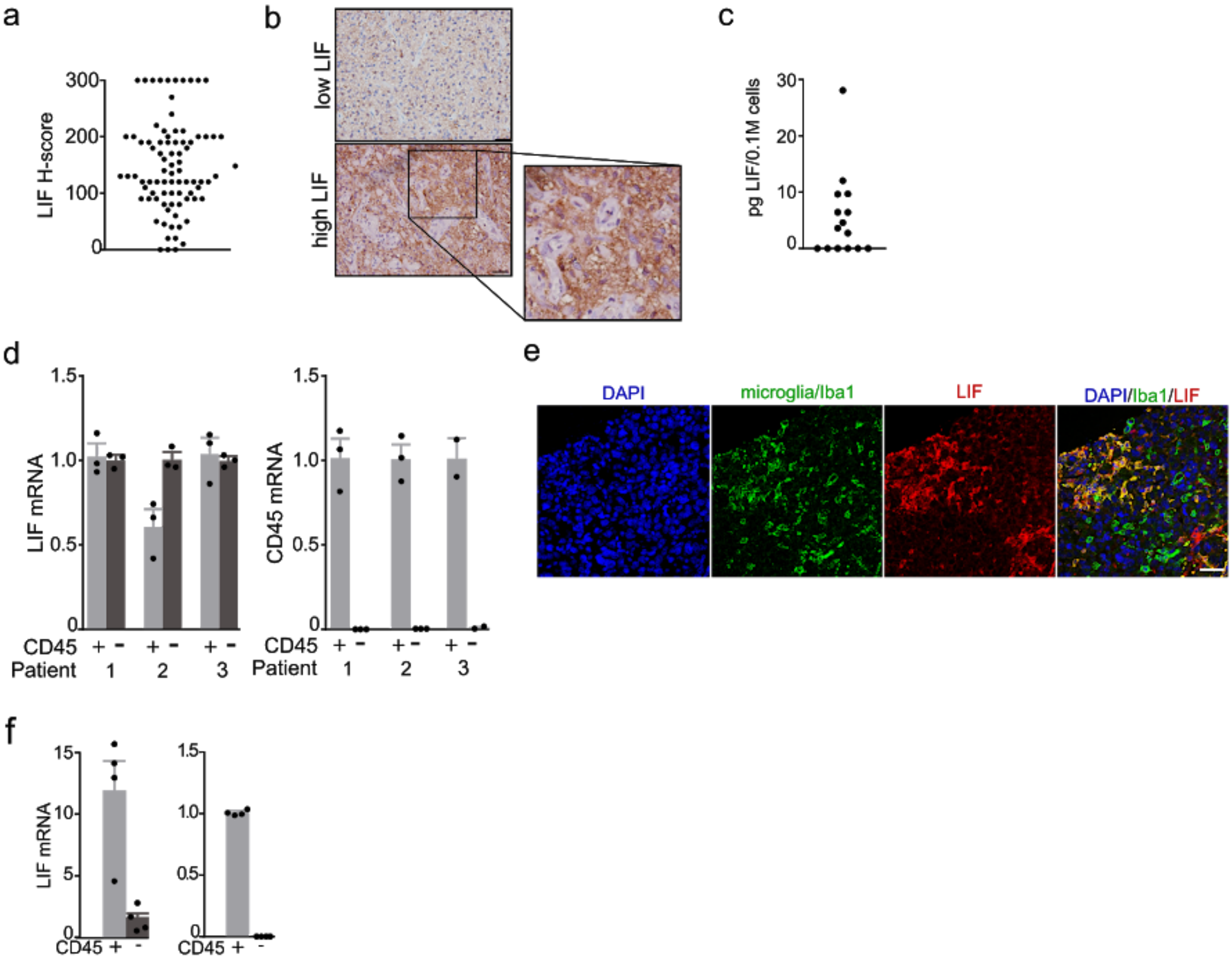

Supplementary Figure 1. LIF expression in tumors.

a, b, LIF IHC was performed in tissue microarrays from human GBM and the degree of staining was quantified using the H-score method (**a**). Representative images are shown. Scale bar, 50 µm (**b**). **c,** LIF ELISA of the supernatant from 15 patient tumoroid cultures. **d, f,** qRT-PCR analyses of the indicated genes in $CD45^{+}$ and $CD45^{-}$ cells isolated from human GBMs (**d**) and GL261N tumors (**f**). **e,** Representative images of IF of Iba1 and LIF counterstained with DAPI in GL261N tumors. Scale bar, 20 µm. Data are presented as mean ± SEM.

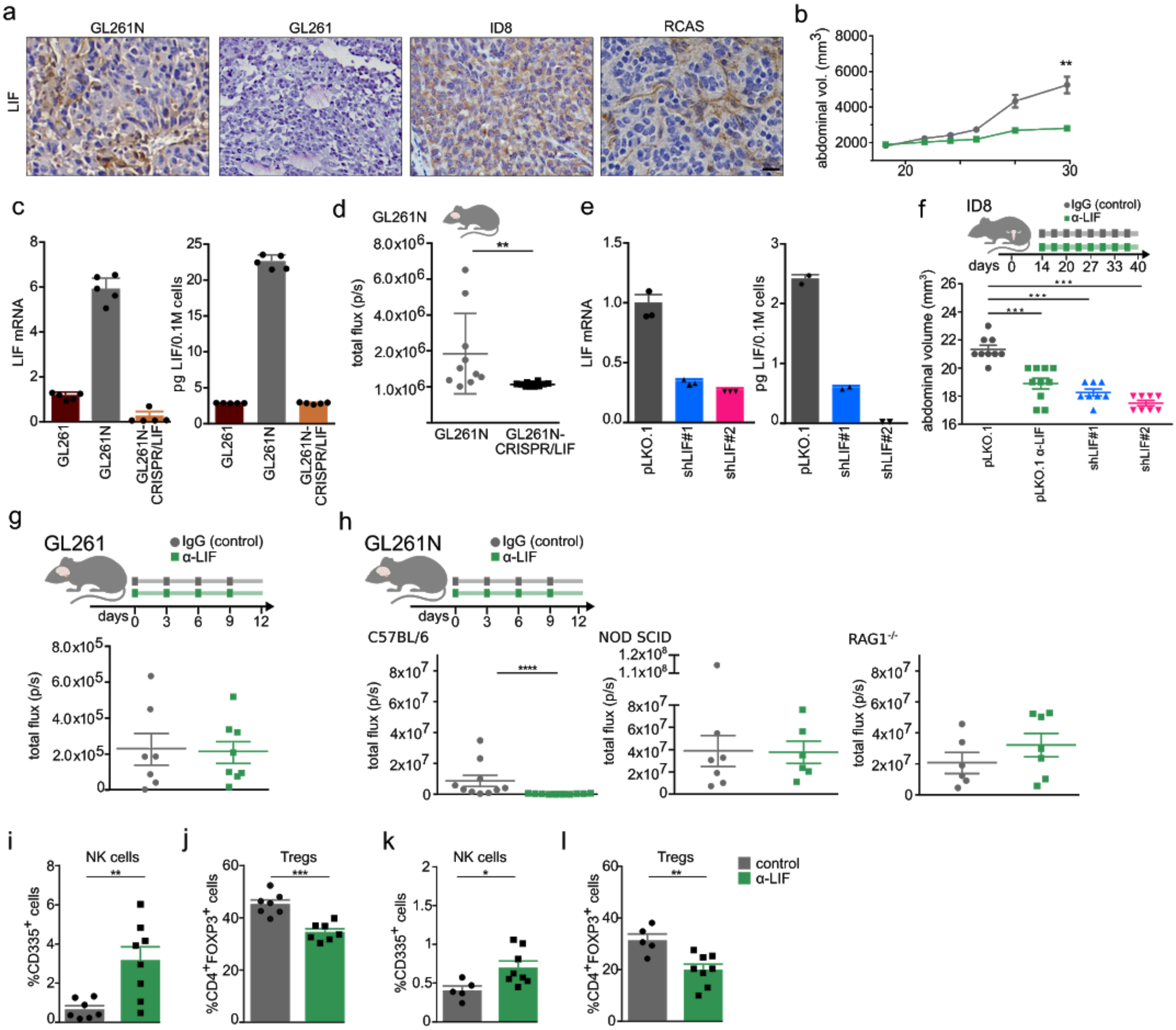

Supplementary Figure 2. LIF blockade in syngeneic models inhibits tumor growth.

a, LIF IHC was performed in GL261N, GL261, ID8, and RCAS tumors. Representative images are shown. Scale bar, 20 µm. **b,** Time course of abdominal volume of ID8 mice treated with anti-LIF or IgG. **c,** qRT-PCR and ELISA of LIF was performed in GL261, GL261N and GL261N-CRISPR/LIF cells. **d,** Tumor growth as total flux (p/s) is shown at 12 dpi in mice inoculated with GL261N and

GL261N-CRISPR/LIF. **e, f,** ID8 cells were infected with the pLKO.1 or two independent pLKO.1-shLIF lentivirus. LIF expression was determined by qRT-PCR and ELISA (**e**). **f,** ID8 cells were inoculated in the peritoneum of mice. Treatment scheme is shown. Abdominal volume (mm^3) was measured at 40 dpi. **g,** GL261 tumor growth in mice treated with anti-LIF or control IgG at 12 dpi. **h,** GL261N cells were inoculated in C57BL/6 mice and two immunodeficient models, NOD SCID and $RAG1^{-/-}$. Treatment scheme is shown. Tumor growth was measured at 12 dpi. **i-l,** Percentages of NK cells ($CD335^+$) and Tregs ($CD3^+CD4^+FoxP3^+$), gated on $CD4^+$ T cell population, in GL261N (**i-j**) and ID8 (**k-l**) tumors were determined by flow cytometry. Data are presented as mean ± SEM. Statistical analysis by Mann-Whitney T test. *P < 0.05; **P < 0.01;***P < 0.001; ****P < 0.0001.

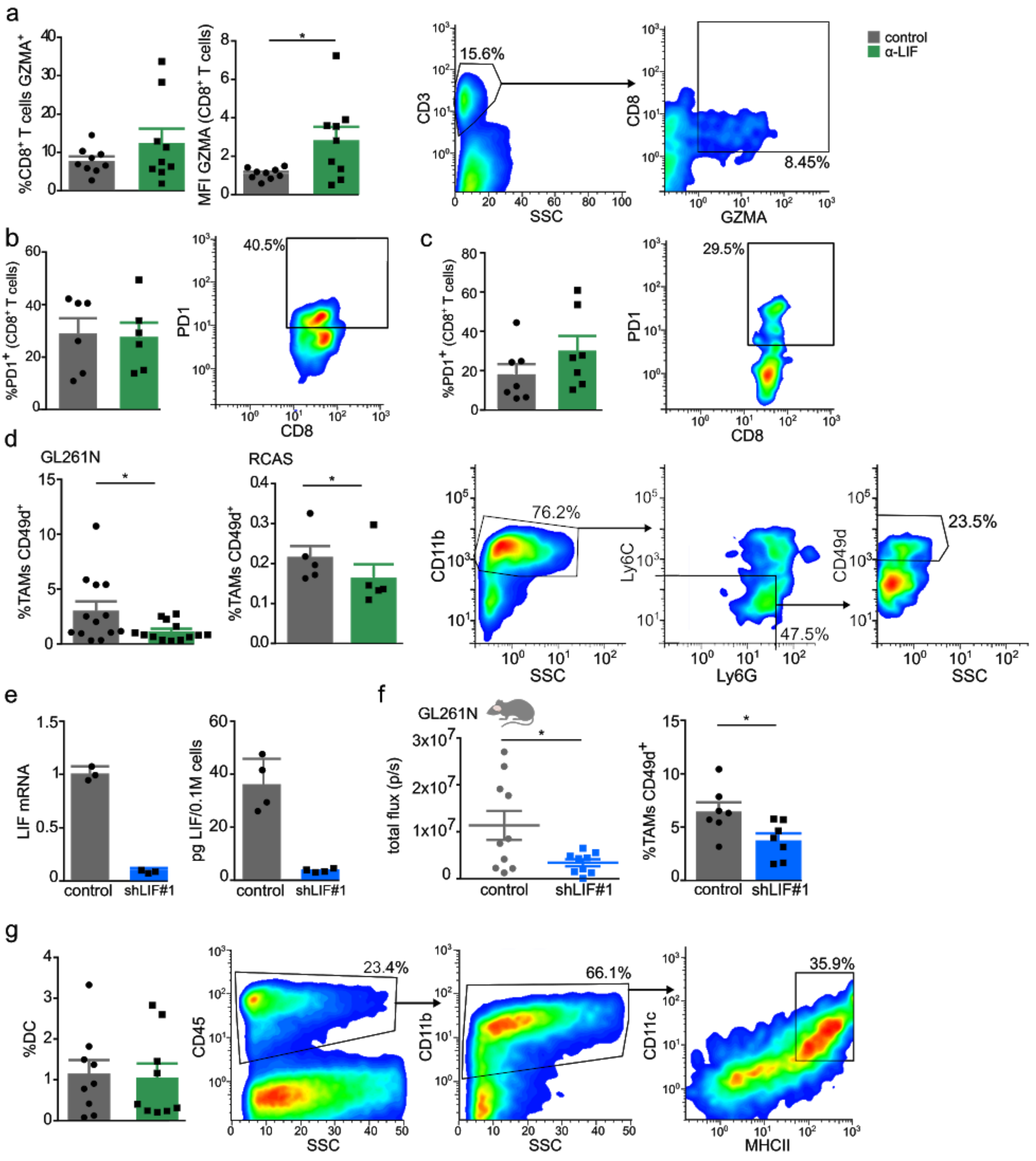

a
%CD8+ T cells GZMA+
MFI GZMA (CD8+ T cells)
15.6%
CD3
SSC
CD8
GZMA
8.45%
control
α-LIF
b
%PD1+ (CD8+ T cells)
40.5%
PD1
CD8
c
29.5%
d
GL261N
%TAMs CD49d+
RCAS
76.2%
CD11b
Ly6C
Ly6G
47.5%
CD49d
23.5%
e
LIF mRNA
pg LIF/0.1M cells
control
shLIF#1
f
total flux (p/s)
g
%DC
23.4%
CD45
66.1%
CD11b
CD11c
MHCII
35.9%

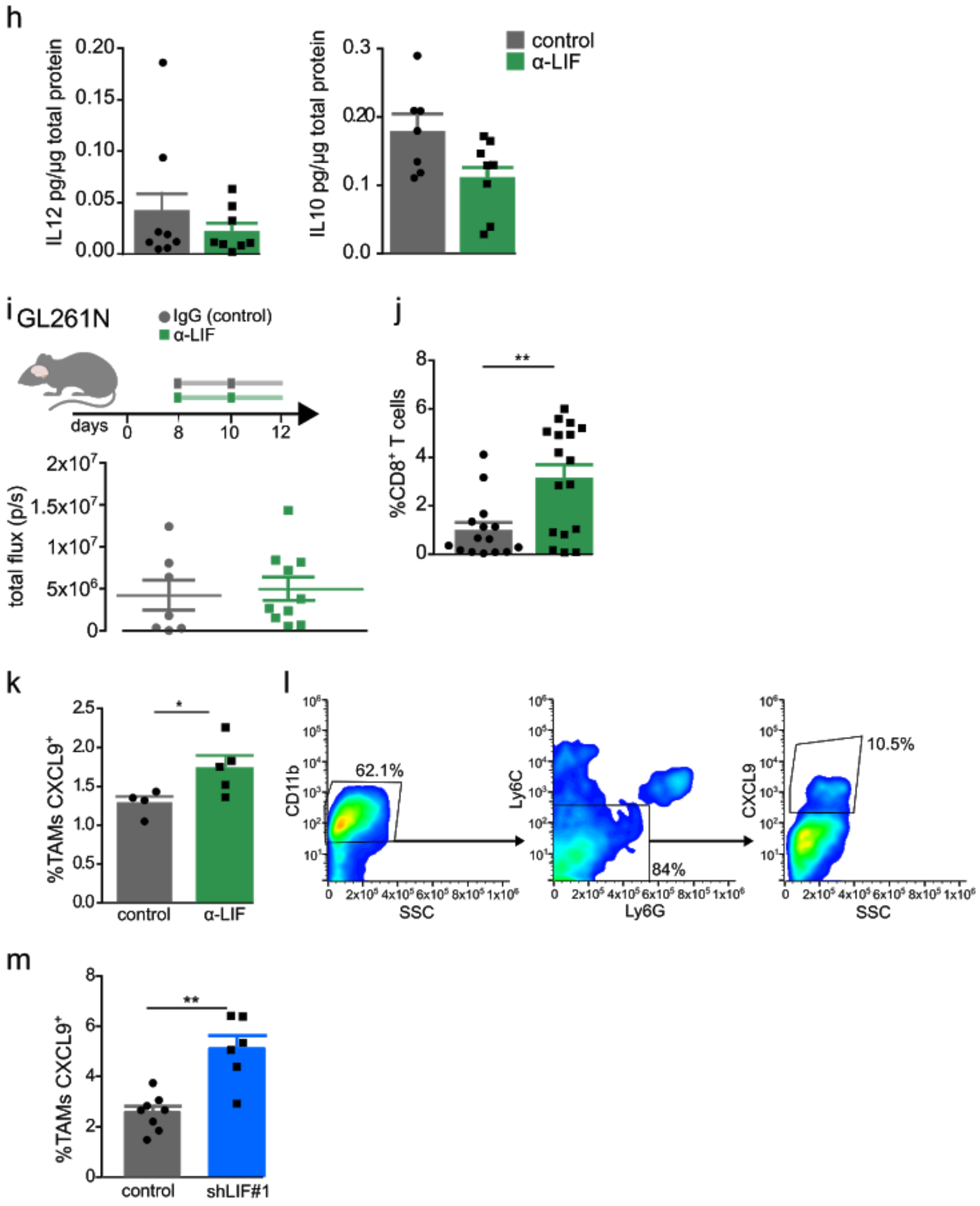

Supplementary Figure 3. Characterization of the immune cell infiltrates upon treatment with anti-LIF.

a, Percentage and MFI of GZMA in the $CD8^+$ T cell population of GL261N tumors. **b, c,** Quantification of $PD1^+$ $CD8^+$ T cells in tumors of GL261N (**b**) and ID8 (**c**) models. **d, f,** Percentage of infiltrating TAMs ($CD11b^+$ $Ly6G^-$ $Ly6C^-$ $CD49d^+$) in GL261N and RCAS tumors in response to treatment with anti-LIF (**d**) or in GL261N shLIF tumors (**f**). **e,** GL261N cells were infected with the

non-specific shRNA (Control) and the shLIF (shLIF#1) lentivirus. LIF expression was determined by qRT-PCR and ELISA. **f,** Tumor growth as total flux (p/s) is shown at 12 dpi in mice inoculated with GL261N control and shLIF#1. **g,** Dendritic cell population (DC) ($CD11b^+$ $CD11c^+$ $MHCII^+$) in GL261N tumors. **a-d, g,** Representative flow cytometry dot plots are shown. **h,** ELISA of IL12 and IL10 in GL261N tumors. **i,** GL261N tumor-bearing mice were treated with anti-LIF at 8 dpi. Treatment scheme is shown. Tumor volume was measured as total flux (p/s) at 13 dpi. **j,** Percentage of $CD8^+$ T cells ($CD3^+$ $CD8^+$) in tumors was determined by flow cytometry. **k**, **m,** Percentage of $CXCL9^+$ in TAMs ($CD11b^+$ $Ly6G^-$ $Ly6C^-$) from anti-LIF treated or untreated RCAS tumors (**k**) or from control or shLIF GL261N tumors (**m**). **l,** Representative flow cytometry dot plots of CXCL9, gated on TAMs, in GL261N tumors. Data are presented as mean ± SEM. Statistical analysis by Mann-Whitney T test. *P < 0.05; **P < 0.01.

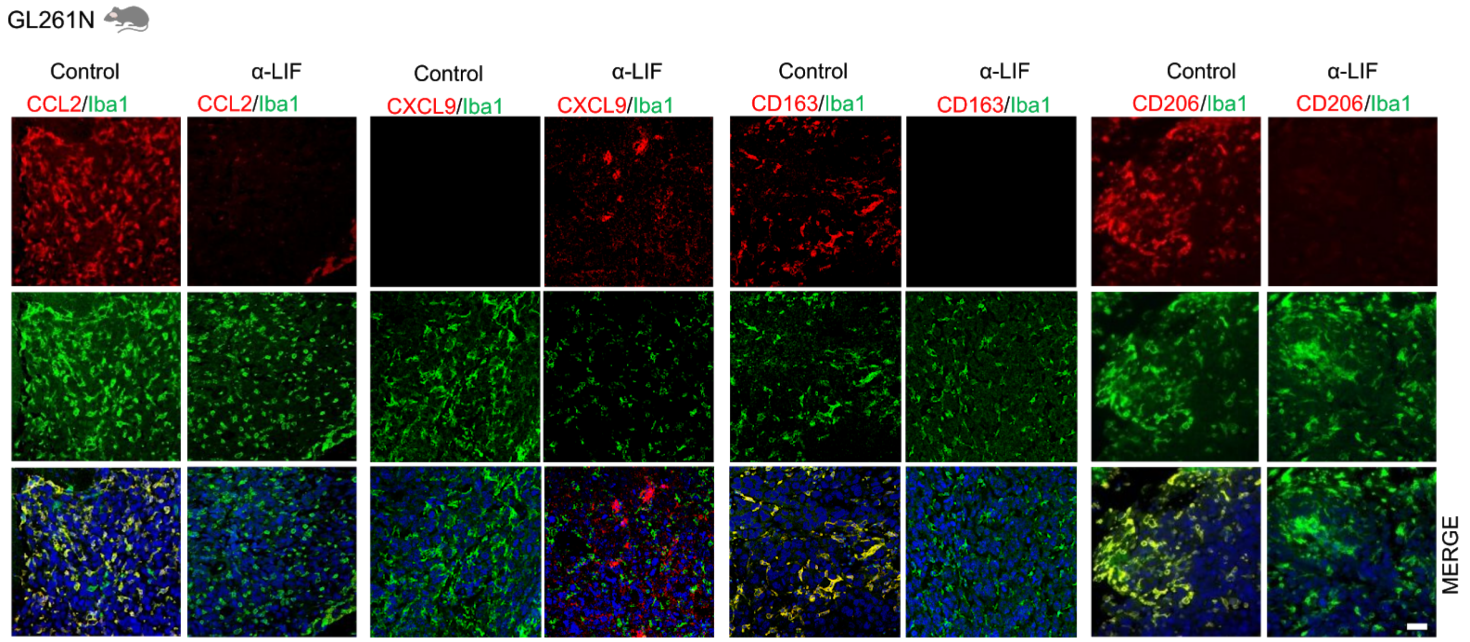

Supplementary Figure 4. Regulation of CXCL9, CCL2, CD163 and CD206 expression by LIF in GL261N tumors.

Individual IF staining images of the indicated factors in GL261N tumors are shown. Scale bar, 20 μm.

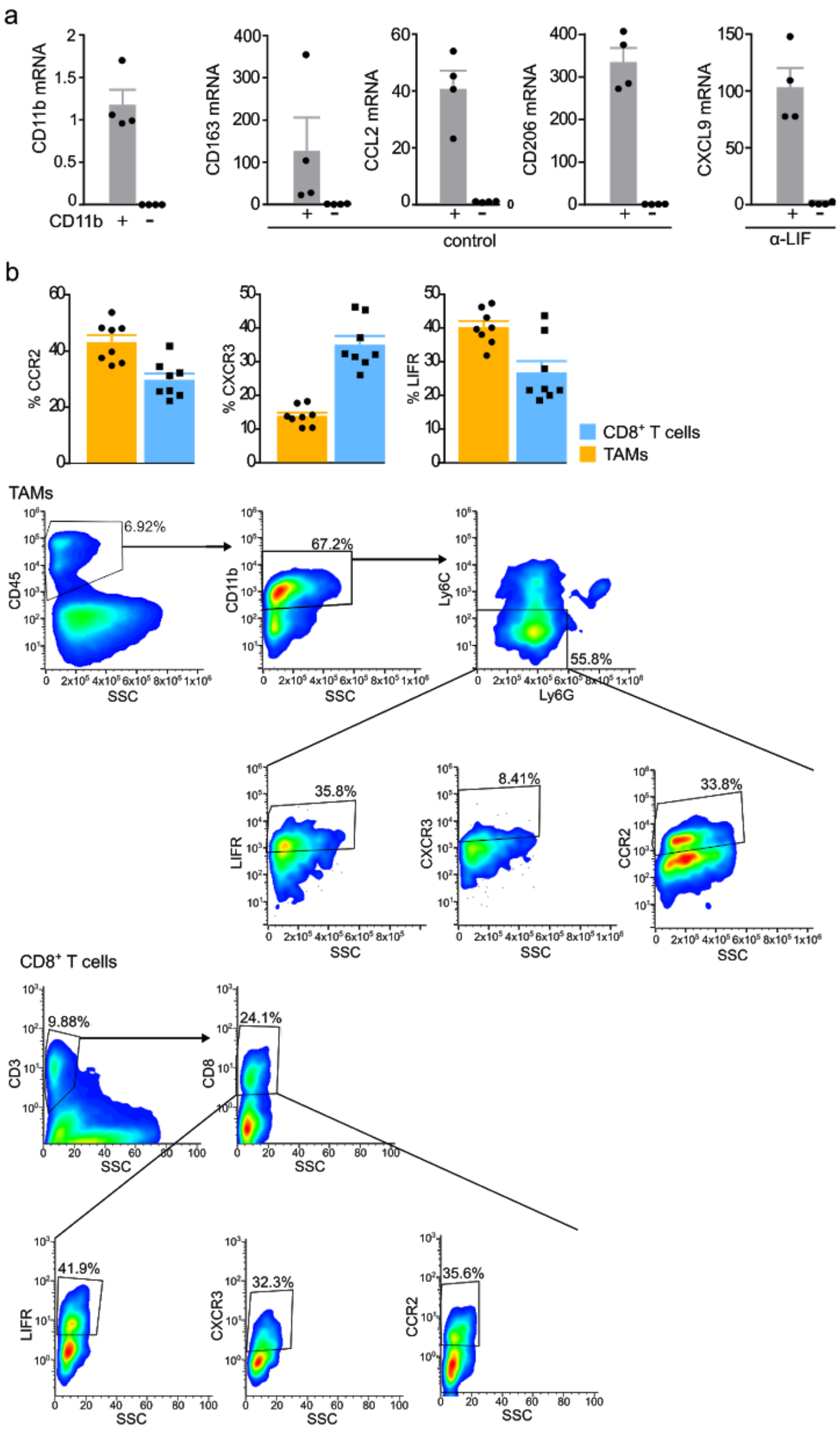
a
CD11b mRNA
CD163 mRNA
CCL2 mRNA
CD206 mRNA
CXCL9 mRNA
CD11b + -
control
α-LIF
b
% CCR2
% CXCR3
% LIFR
CD8⁺ T cells
TAMs
CD45
CD11b
Ly6C
Ly6G
SSC
6.92%
67.2%
55.8%
LIFR
CXCR3
CCR2
35.8%
8.41%
33.8%
CD3
CD8
9.88%
24.1%
41.9%
32.3%
35.6%

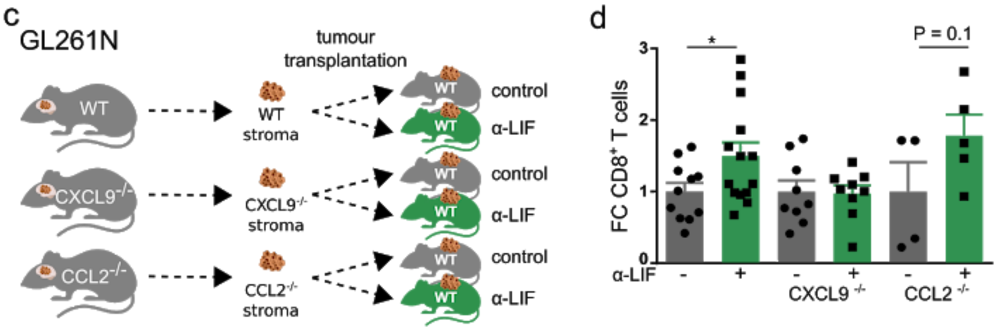

Supplementary Figure 5. Cell specific expression of cytokines and receptors, and functional contributions.

a, qRT-PCR analyses of the indicated genes in CD11b$^+$ Ly6G$^-$ Ly6C$^-$ and CD11b$^-$ Ly6G$^-$ Ly6C$^-$ cells sorted from GL261N tumors. **b,** Percentage of CCR2, CXCR3 and LIFR receptors in TAMs (CD11b$^+$ Ly6G$^-$ Ly6C$^-$) and CD8$^+$ T cell (CD3$^+$ CD8$^+$) populations determined by flow cytometry. Representative flow cytometry dot plots are shown. **c, d,** GL261N tumors from WT, CXCL9$^{-/-}$ and CCL2$^{-/-}$ mice were isolated and subcutaneously implanted into control or anti-LIF treated mice. Schematic representation of the experimental procedures is shown (**c**). **d,** 48 h after transplantation, FC of infiltrating CD8$^+$ T cells were analysed by flow cytometry. Data are presented as mean ± SEM. Statistical analysis by Mann-Whitney T test. *P < 0.05.

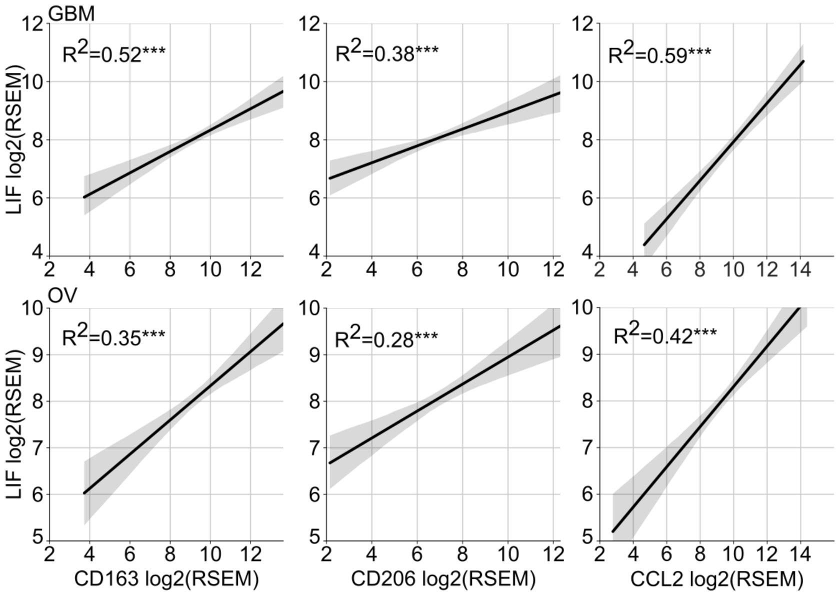

Supplementary Figure 6. Correlation between LIF and CD163, CD206 and CCL2 expression in GBM and ovarian cancer.

Regression plots between LIF and CD163, CD206, CCL2 expression (in log2 RSEM) in GBM and ovarian cancer (OV) TCGA tumor cohorts (see Supplementary Data 1b). The shade represents the confidence intervals of the regression estimate.

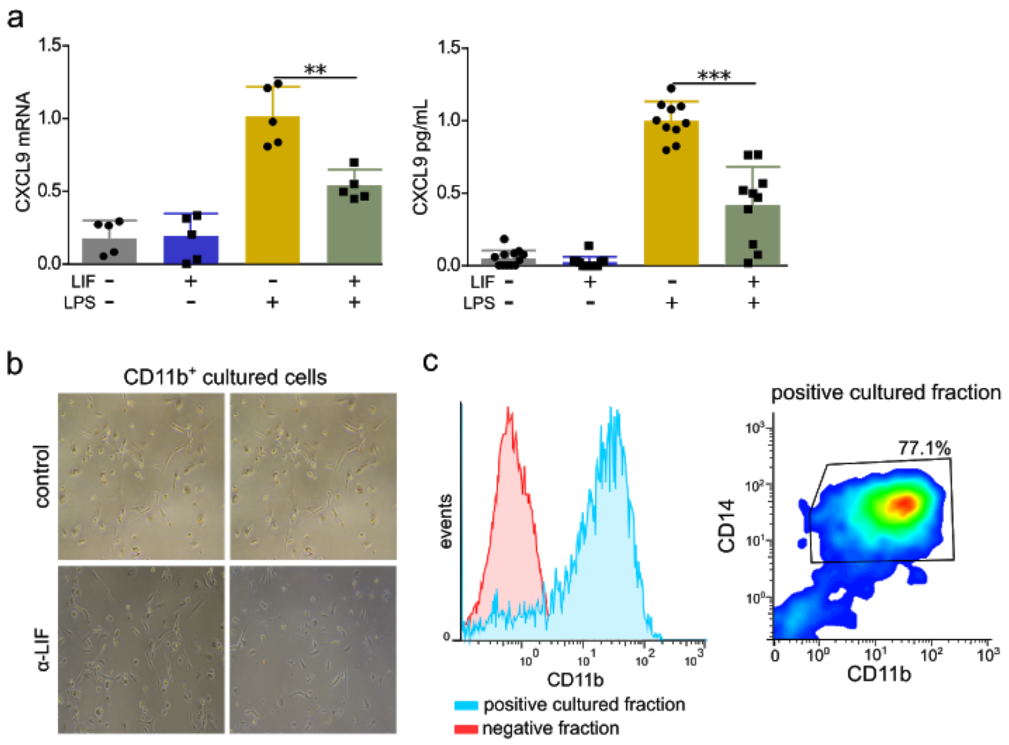

Supplementary Figure 7. Regulation of CXCL9 by LIF in murine and human macrophages.

a, BMDMs were pre-incubated with 20 ng/ml LIF for 18 h and then stimulated with 1 μg/ml LPS for 6 h. *CXCL9* mRNA expression and secretion were determined by qRT-PCR and ELISA, respectively. **b, c,** $CD11b^+$ sorted cells from human GBM were cultured. Representative images of control and treated cells are shown (**b**). Percentage of $CD11b^+$ $CD14^+$ cells in the culture was determined by flow cytometry. Data are presented as mean ± SD. Statistical analyses by Student's *t*-test. $^{**}P < 0.01$; $^{***}P < 0.001$.

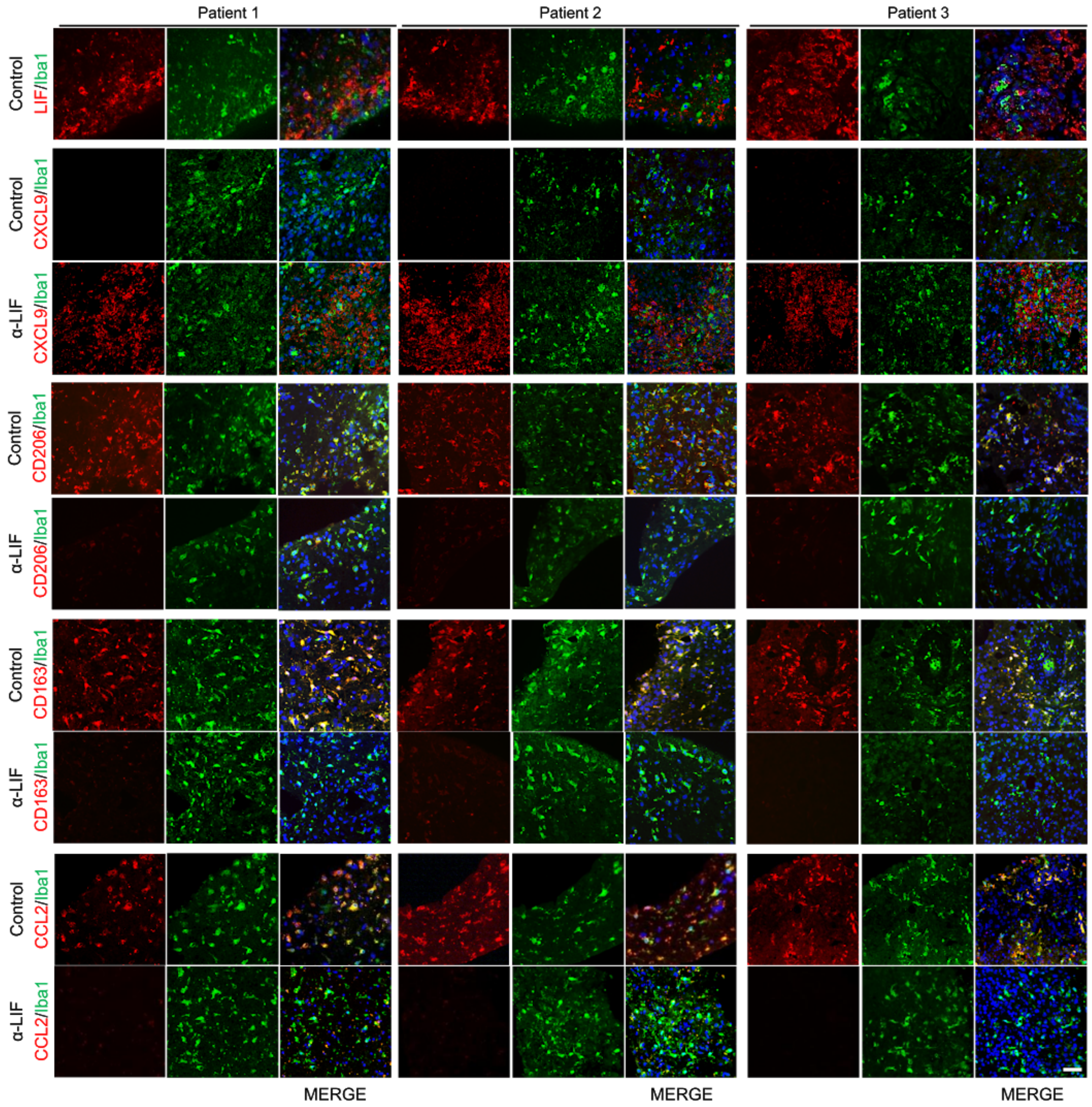

Supplementary Figure 8. Regulation of CXCL9, CCL2, CD163 and CD206 expression by LIF in human GBM tumors.

Individual IF staining images of the indicated factors in human GBM organotypic slice cultures are shown. Scale bar, 20 µm.

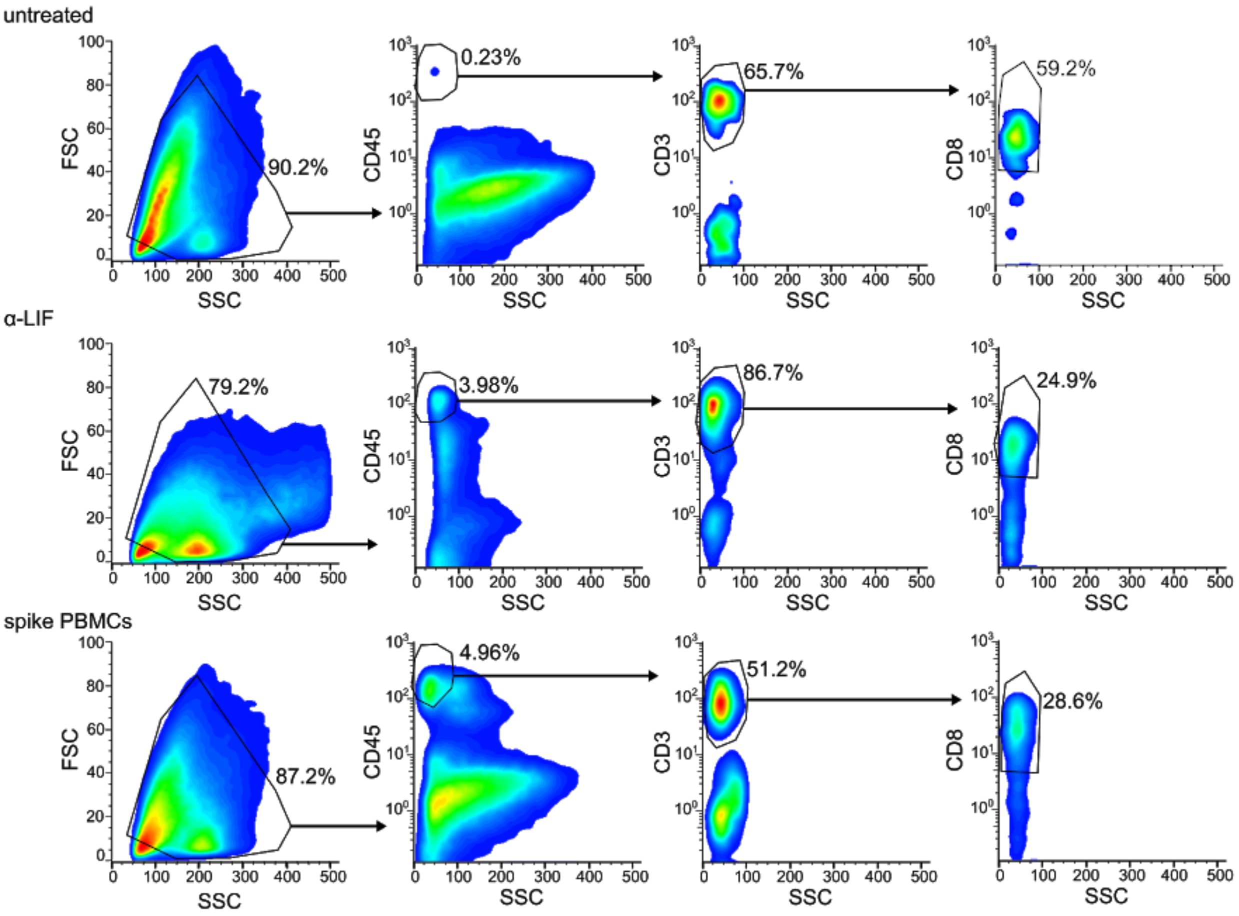

Supplementary Figure 9. LIF regulates infiltration of CD8^{+} T cells into human GBM organotypic slice cultures.

Representative flow cytometry plots of untreated, anti-LIF or positive control (spike PBMCs) of human GBM organotypic specimens cultured with PBMCs for 48 h. Percentage of CD8^{+} T cells was determined within CD3^{+} cells within the CD45^{+} population.

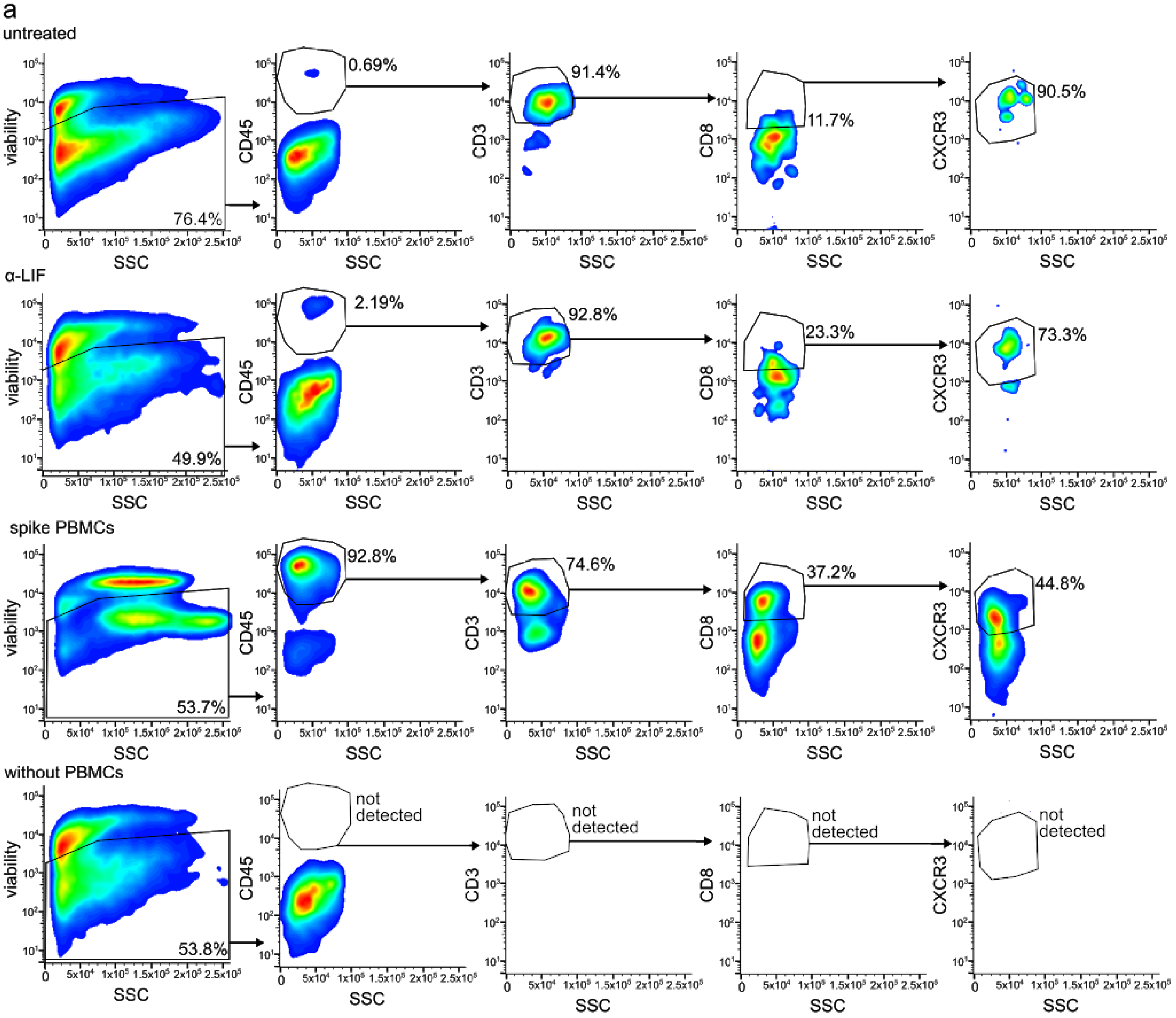

a
untreated
α-LIF
spike PBMCs
without PBMCs
viability
CD45
CD3
CD8
CXCR3
SSC
76.4%
0.69%
91.4%
11.7%
90.5%
49.9%
2.19%
92.8%
23.3%
73.3%
53.7%
92.8%
74.6%
37.2%
44.8%
53.8%
not detected
not detected
not detected
not detected

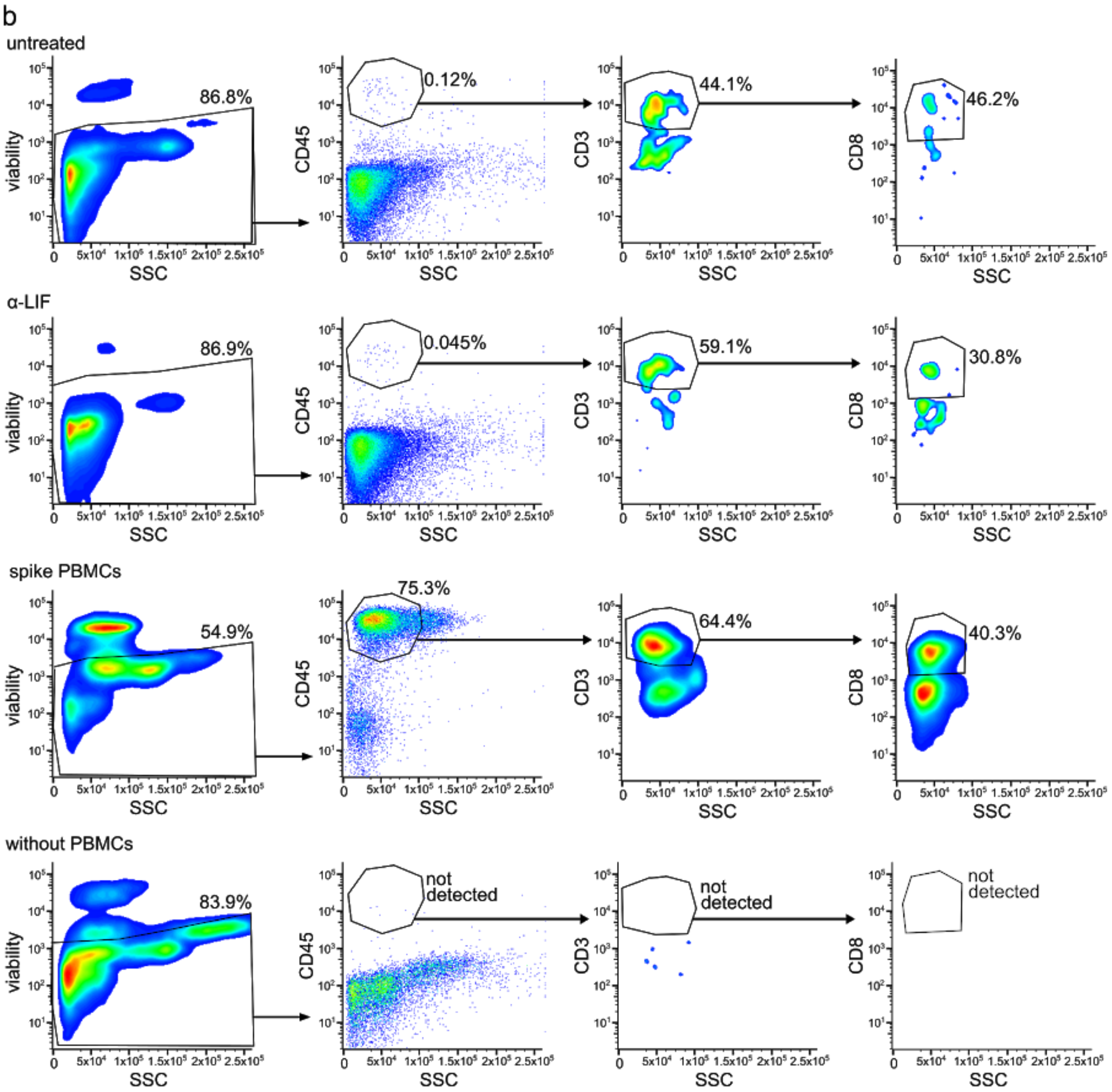
b
untreated
86.8%
0.12%
44.1%
46.2%
α-LIF
86.9%
0.045%
59.1%
30.8%
spike PBMCs
54.9%
75.3%
64.4%
40.3%
without PBMCs
83.9%
not detected
not detected
not detected
viability
CD45
CD3
CD8
SSC

c

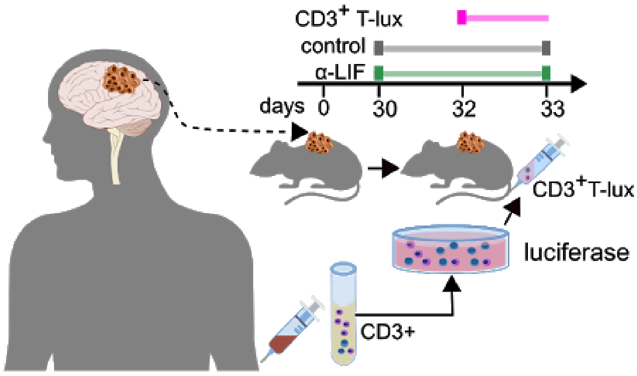

d

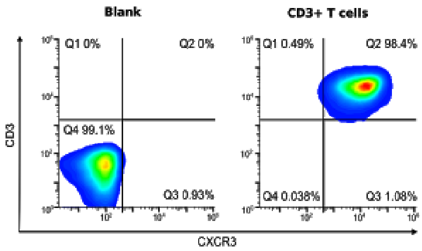

Supplementary Figure 10. LIF regulates infiltration of CD8$^+$ T cells into human GBM specimens *in vivo*.

a, b, Representative flow cytometry plots of tumors (**a**) or blood (**b**) obtained from untreated or anti-LIF treated human GBM tumors engrafted into NSG mice and inoculated with human PBMCs. Positive control (spike PBMCs) and negative control (no PBMCs inoculation) are included. Percentage of CD8$^+$ T cells was determined within CD3$^+$ cells within the CD45$^+$ population. A marker of viability was also used. In some occasions CXCR3 (CXCL9 receptor) was also determined inside the CD8$^+$ T cell population. Importantly, a negative control (without PBMCs) was also used to assure specificity of leukocyte detection. **c,** Schematic representation of the experimental procedure performed by inoculating CD3$^+$ T cells expressing luciferase (CD3$^+$T-lux) into mice bearing human GBM tumors. **d,** Flow cytometry plots representing percentage of CXCR3$^+$ cells in luciferase-expressing CD3$^+$ T cells.

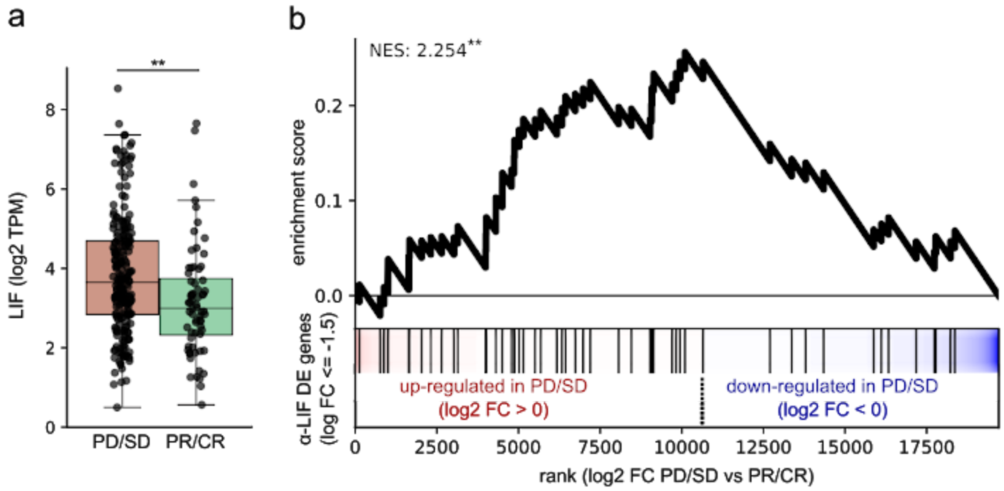

Supplementary Figure 11. LIF is associated to anti-PD1 treatment resistance in clinical trials.

a, LIF is up-regulated in non-responder anti-PD1 treated patients. Boxplot representing LIF differential expression between non-responder anti-PD1 treated patients (PD/SD: Progressive Disease/Sustained Disease) and responder patients (PR/CR: Partial response/Complete Response). Middle line depicts the median and the whiskers the interquartile range. Statistical analysis by Mann-Whitney T test. **P < 0.01. **b,** Genes down-regulated upon anti-LIF treatment are up-regulated in anti-PD1 trated non-responder patients. Enrichment plot of the Gene Set Enrichment (GSEA) analysis of the genes down-regulated upon anti-LIF treatment in non-responder tumors. Genes down-regulated upon anti-LIF treatment were obtained from the list of genes differentially expressed in anti-LIF treated ID8 mice; all genes with a log Fold Change (FC) < -1.5 were considered (see Supplementary Data 1). NES (normalised enrichment score). Statistical analysis by GSEA permutation test (n=10,000). **P < 0.01.
