# Supplementary material for: LIF regulates CXCL9 in tumor-associated macrophages and prevents CD8+ T cell tumor-infiltration impairing anti-PD1 therapy
Source: Nat Commun. 2019 Jun 11;10:2416. doi: 10.1038/s41467-019-10369-9 (PMC6559950; doi:10.1038/s41467-019-10369-9)
Supplement: Supplementary file 4 — Reporting Summary [file 41467_2019_10369_MOESM4_ESM.pdf]

nature research

Corresponding author(s): Joan Seoane

Last updated by author(s): Apr 24, 2019

Reporting Summary

Nature Research wishes to improve the reproducibility of the work that we publish. This form provides structure for consistency and transparency in reporting. For further information on Nature Research policies, see Authors & Referees and the Editorial Policy Checklist.

Statistics

For all statistical analyses, confirm that the following items are present in the figure legend, table legend, main text, or Methods section.

n/a	Confirmed	
☐	☒	The exact sample size (*n*) for each experimental group/condition, given as a discrete number and unit of measurement
☐	☒	A statement on whether measurements were taken from distinct samples or whether the same sample was measured repeatedly
☐	☒	The statistical test(s) used AND whether they are one- or two-sided<br>*Only common tests should be described solely by name; describe more complex techniques in the Methods section.*
☐	☒	A description of all covariates tested
☐	☒	A description of any assumptions or corrections, such as tests of normality and adjustment for multiple comparisons
☐	☒	A full description of the statistical parameters including central tendency (e.g. means) or other basic estimates (e.g. regression coefficient) AND variation (e.g. standard deviation) or associated estimates of uncertainty (e.g. confidence intervals)
☐	☒	For null hypothesis testing, the test statistic (e.g. *F*, *t*, *r*) with confidence intervals, effect sizes, degrees of freedom and *P* value noted<br>*Give P values as exact values whenever suitable.*
☐	☒	For Bayesian analysis, information on the choice of priors and Markov chain Monte Carlo settings
☐	☒	For hierarchical and complex designs, identification of the appropriate level for tests and full reporting of outcomes
☐	☒	Estimates of effect sizes (e.g. Cohen's *d*, Pearson's *r*), indicating how they were calculated

Our web collection on statistics for biologists contains articles on many of the points above.

Software and code

Policy information about availability of computer code

Data collection	no custom software was used
Data analysis	no custom software was used

For manuscripts utilizing custom algorithms or software that are central to the research but not yet described in published literature, software must be made available to editors/reviewers. We strongly encourage code deposition in a community repository (e.g. GitHub). See the Nature Research guidelines for submitting code & software for further information.

Data

Policy information about availability of data

All manuscripts must include a data availability statement. This statement should provide the following information, where applicable:

- Accession codes, unique identifiers, or web links for publicly available datasets
- A list of figures that have associated raw data
- A description of any restrictions on data availability

Data have been deposited in the Gene Expression Omnibus (GEO)/NCBI public database (accession no. GSE79852);
Fig1-Fig4;
no restrictions on data availability

Field-specific reporting

Please select the one below that is the best fit for your research. If you are not sure, read the appropriate sections before making your selection.

☒ Life sciences ☐ Behavioural & social sciences ☐ Ecological, evolutionary & environmental sciences

For a reference copy of the document with all sections, see nature.com/documents/nr-reporting-summary-flat.pdf

Life sciences study design

All studies must disclose on these points even when the disclosure is negative.

Sample size	In vivo experiments were performed with 8 to 10 animals per point. This sample size provides statistically meaningful results.
Data exclusions	No data was excluded
Replication	The experiments were repeated at least three times
Randomization	Animals were allocated into different experimental groups randomly
Blinding	Blinding was not relevant in our experiments

Reporting for specific materials, systems and methods

We require information from authors about some types of materials, experimental systems and methods used in many studies. Here, indicate whether each material, system or method listed is relevant to your study. If you are not sure if a list item applies to your research, read the appropriate section before selecting a response.

Materials & experimental systems

n/a	Involved in the study	
☐	☒	Antibodies
☐	☒	Eukaryotic cell lines
☒	☐	Palaeontology
☐	☒	Animals and other organisms
☐	☒	Human research participants
☒	☐	Clinical data

Methods

n/a	Involved in the study	
☒	☐	ChIP-seq
☐	☒	Flow cytometry
☒	☐	MRI-based neuroimaging

Antibodies

Antibodies used	Antibodies against human LIF (Atlas; 1:200), murine LIF (AbCam; 1:200), p-STAT3 (Cell Signaling; 1:50), Ki67 (AbCam; 1:200), Cleaved-Caspase3 (CC3) (Cell Signaling; 1:500), CD4 (Leica; 1:100), murine CD8 (Bioss; 1:200), CCL2 (Novus Biologicals, 1:200), FoxP3 (Novus Biologicals; 1:50), and human CD163 (Leica Novacastra; 1:200) were used for immunohistochemical stainings. Antibodies against CCL2 (Novus Biologicals, 1:200), CD11b (AbCam; 1:2000), IBA-1 (Wako; 1:1000), murine CD68 (AbCam; 1:200), CD3 (Leica;1:500) FoxP3 (Novus Biologicals, 1:50), CD206 (Abcam; 1:500), murine CD163 (Abcam; 1:200), CXCL9 (Thermo Fischer Scientific; 1:100) and human CD8 (DAKO; 1:200) were used for immunofluorescence. The antibodies against CD3, CD4, CD335, CD163, MHC class II (eBioscience), CD8, F4/80, CD11b, and CD206 (BD Bioscience) and PD1 (Biolegend) were used for flow cytometry.
Validation	All antibodies were used and validated in previous publications

Eukaryotic cell lines

Policy information about cell lines

Cell line source(s)	GL261N were derived from GL261 cells that were obtained from Charles River and cultured in RPMI (Life Technologies). ID8 was a generous gift from Dr. George Coukos, Ludwing Institute for Cancer Research, Lausanne.
Authentication	The cell lines were not authenticated
Mycoplasma contamination	All cell lines were tested negative for mycoplasma
Commonly misidentified lines (See ICLAC register)	N/A

Animals and other organisms

Policy information about studies involving animals; ARRIVE guidelines recommended for reporting animal research

Laboratory animals	mice, C57/BL6, female, 7 weeks old
Wild animals	N/A
Field-collected samples	N/A
Ethics oversight	All animal experiments were approved by and performed according to the guidelines of the Institutional Animal Care Committee of the Vall d'Hebron Research Institute in agreement with the European Union and national directives.

Note that full information on the approval of the study protocol must also be provided in the manuscript.

Human research participants

Policy information about studies involving human research participants

Population characteristics	N/A
Recruitment	N/A
Ethics oversight	Human GBM specimens were obtained from the Vall d'Hebron University Hospital and Clinic Hospital. The clinical protocol was approved by the Vall d'Hebron Institutional Review Board and Clinic Hospital (CEIC), with informed consent obtained from all subjects.

Note that full information on the approval of the study protocol must also be provided in the manuscript.

Flow Cytometry

Plots

Confirm that:

☒ The axis labels state the marker and fluorochrome used (e.g. CD4-FITC).

☒ The axis scales are clearly visible. Include numbers along axes only for bottom left plot of group (a 'group' is an analysis of identical markers).

☒ All plots are contour plots with outliers or pseudocolor plots.

☒ A numerical value for number of cells or percentage (with statistics) is provided.

Methodology

Sample preparation	Mice were euthanized and tumours were isolated. GL261N tumours were enzymatically digested with Brain Tumor Dissociation kit and myelin was removed with Myelin Removal Beads II (all from Miltenyi Biotec). ID8 tumours were processed with Mouse Tumor Dissociation kit (Miltenyi Biotec) and ascitic liquids were collected. CD11b+ cells isolation from GL261N and ID8 cell suspension was performed with CD11b magnetic beads using the MultiMACS Cell24 separator Plus (all from Miltenyi Biotec), following manufacturer instructions. The antibodies against CD3, CD4, CD335, CD163, MHC class II (eBioscience), CD8, F4/80, CD11b, and CD206 (BD Bioscience) and PD1 (Biolegend) were used for flow cytometry using the BD LSRFortessa™ cell analyzer, and data were analyzed with Flow Jo software. Intracellular staining of FoxP3 (eBioscience) were performed using a specific staining set (eBioscience).
Instrument	BD LSRFortessa™ cell analyzer
Software	Flow Jo software
Cell population abundance	No sorting was performed
Gating strategy	Preliminary FSC/SSD gates were always performed and the negative/positive boundaries were obtained using negative controls such as abscence of primary Ab.

☒ Tick this box to confirm that a figure exemplifying the gating strategy is provided in the Supplementary Information.
