# Supplementary material for: LIF regulates CXCL9 in tumor-associated macrophages and prevents CD8+ T cell tumor-infiltration impairing anti-PD1 therapy
Source: Nat Commun. 2019 Jun 11;10:2416. doi: 10.1038/s41467-019-10369-9 (PMC6559950; doi:10.1038/s41467-019-10369-9)
Supplement: Supplementary file 5 — Description of Additional Supplementary Files [file 41467_2019_10369_MOESM5_ESM.docx]

## Title: Supplementary Data 1. Description of *in silico* immune infiltration datasets.

**Description:** (associated to Figure 1a and b)

Supplementary Data 1a. Gene signature used to detect the relative infiltration of TAMs.

Supplementary Data 1b. Summary of the TCGA tumor cohorts employed in the study, including the number of samples considered for each cohort, the cancer type acronym used in the figures and its corresponding full name.

Supplementary Data 1c. Pearson correlation values (named R^2^ in Figure 1) between the relative abundance of TAMs (ssGSEA scores) and LIF expression (log2 RSEM) across cancer types. P-value of the correlation and adjusted P-value (FDR Benjamini Hochberg) are shown.

## Title: Supplementary Data 2. Differential expression analysis in TAMs from anti-LIF treated mice.

**Description:** (associated to Figure 2a)

Results of the differential expression analysis of microarray data from TAMs comparing control and anti-LIF treated ID8 mice. For each gene log2 Fold Change (FC), mean expression across all microarray expression profiles, P-value and adjusted P-value (False Discovery Rate) are shown, all parameters have been determined through *limma* (see Methods). Gene significantly (adjusted P-value < 0.1) over-expressed (FC > 1.5) and under-expressed (FC < -1.5) are highlighted in yellow. In orange are highlighted genes experimentally validated (see Figure 2b to e).

Supplementary Data 2a. Genes over-expressed.

Supplementary Data 2b. Genes under-expressed.
